# Predictions of rhizosphere microbiome dynamics with a genome-informed and trait-based energy budget model

**DOI:** 10.1038/s41564-023-01582-w

**Published:** 2024-02-05

**Authors:** Gianna L. Marschmann, Jinyun Tang, Kateryna Zhalnina, Ulas Karaoz, Heejung Cho, Beatrice Le, Jennifer Pett-Ridge, Eoin L. Brodie

**Affiliations:** 1 Earth and Environmental Sciences, Lawrence Berkeley National Laboratory, Berkeley, CA USA; 2 Environmental Genomics and Systems Biology, Lawrence Berkeley National Laboratory, Berkeley, CA USA; 3 Department of Plant and Microbial Biology, University of California Berkeley, Berkeley, CA USA; 4 Physical and Life Sciences Directorate, Lawrence Livermore National Laboratory, Livermore, CA USA; 5 Life and Environmental Sciences Department, University of California Merced, Merced, CA USA; 6 Department of Environmental Science, Policy and Management, University of California Berkeley, Berkeley, CA USA

**Keywords:** Microbial ecology, Biogeochemistry

## Abstract

Soil microbiomes are highly diverse, and to improve their representation in biogeochemical models, microbial genome data can be leveraged to infer key functional traits. By integrating genome-inferred traits into a theory-based hierarchical framework, emergent behaviour arising from interactions of individual traits can be predicted. Here we combine theory-driven predictions of substrate uptake kinetics with a genome-informed trait-based dynamic energy budget model to predict emergent life-history traits and trade-offs in soil bacteria. When applied to a plant microbiome system, the model accurately predicted distinct substrate-acquisition strategies that aligned with observations, uncovering resource-dependent trade-offs between microbial growth rate and efficiency. For instance, inherently slower-growing microorganisms, favoured by organic acid exudation at later plant growth stages, exhibited enhanced carbon use efficiency (yield) without sacrificing growth rate (power). This insight has implications for retaining plant root-derived carbon in soils and highlights the power of data-driven, trait-based approaches for improving microbial representation in biogeochemical models.

## Abstract (web summary)

A trait-based approach harnesses the vast amount of microbial genomic data and enables integration with biogeochemical models, predicting soil microbial ecology and physiology without the need for cultures.

## Main

Microbes are major drivers of carbon (C) and nutrient fluxes in Earth’s terrestrial ecosystems; however, Earth system models designed to inform climate change adaptation and mitigation strategies have typically not included explicit representation of soil microorganisms, despite mounting evidence that the explicit parameterization of microbial processes improves model prediction and reduces uncertainty in terrestrial systems^1,2^. This lack of representation is rooted in the failure of common organizing principles derived from plant and animal ecology, such as the descendants of r-K selection theory^3^ or Grime’s competition–stress tolerance–ruderal (C–S–R) framework^4,5^, to fully capture the complexity of microbial systems^6–9^. To accurately predict the sensitivities of soil organic C stocks and plant productivity in response to changing climate conditions, it is crucial to develop a more comprehensive understanding of the role of microbes and their traits. However, the majority of organisms comprising Earth’s microbiomes have yet to be cultivated and may never be^10^. This means that our window into the ecology of microorganisms, such as those in soil, remains primarily through the lens of genomic information, which is accumulating far more rapidly than phenotypic information from laboratory isolates^11,12^.

Trait-based models have the potential to represent trait variation by aggregating data from hundreds or thousands of genomes, providing a data-driven approach to organize the complexity of microbial communities with less emphasis on traditional ecological theory and phylogenetic origin^13^. Among the various modelling approaches available for studying microbiomes, trait-based models are an attractive intermediate complexity approach to exploring how the hierarchy of traits interacts to influence the fitness of microorganisms within a community^14^. Yet, despite this promise and potential to scale the representation of microorganisms using a trait-based representation in biogeochemical models, a major challenge remains in their parameterization^15^. Our recent work provides a computational pipeline and a toolset (‘microTrait’^16^) to infer microbial traits from genomic data and establish links between each genome-derived trait and ecological strategy at different levels of trait granularity. The resulting information can be used to initialize and parameterize mechanistic trait-based models spanning a hierarchy of structural complexity to explore the drivers of variation in the distribution and co-occurrence of microbial traits^17^. Moreover, incorporating emerging concepts and theory outside of traditional microbiome science, such as thermodynamic and biophysical theory, proves valuable for understanding traits of microorganisms^18^. This integration facilitates the search for generalizable principles or ‘rules’ that are applicable across diverse microbiome systems^19^, furthering theory development, and the elaboration of large-scale predictive models^15^.

**Fig. 1:**
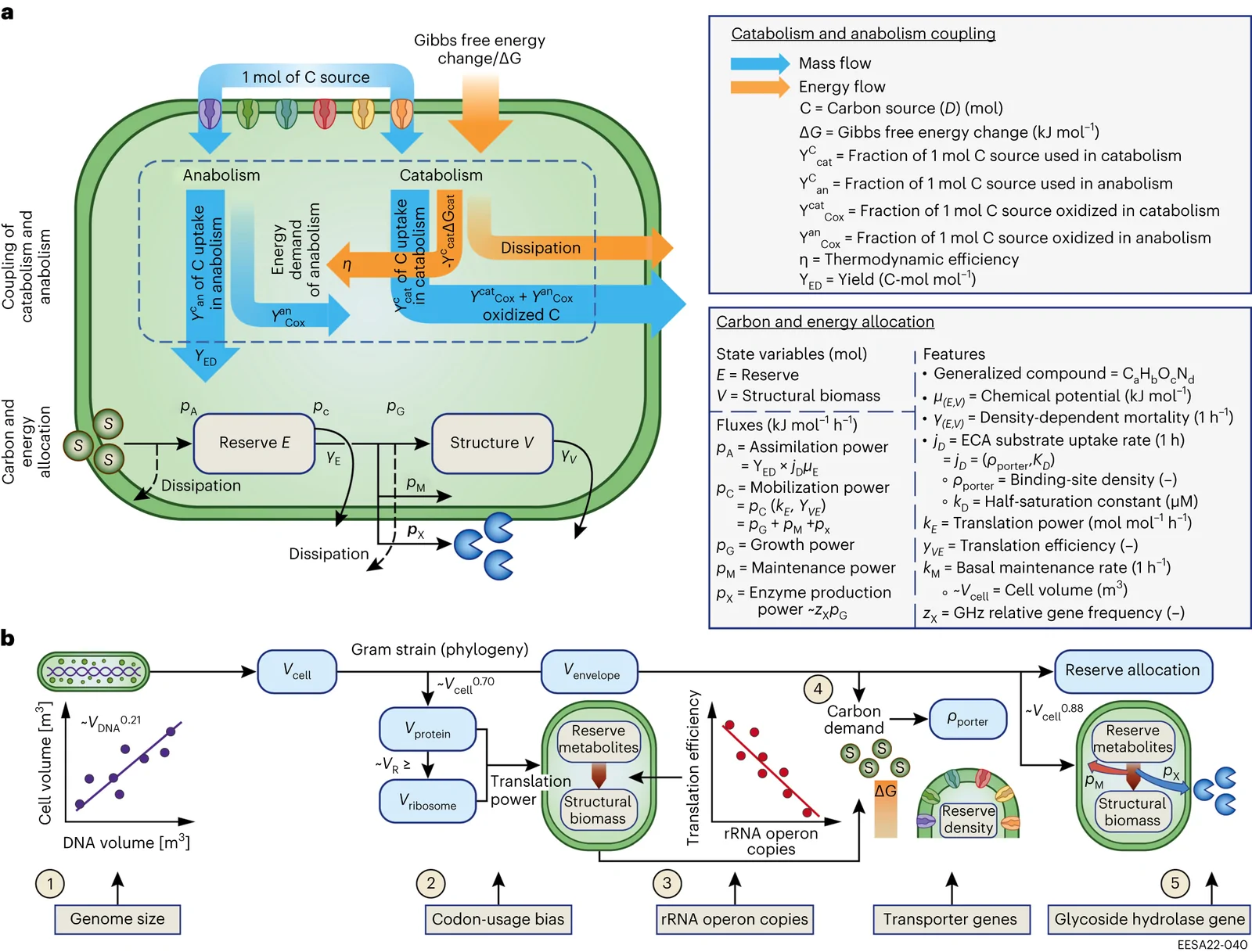
Overview of DEBmicroTrait. **a**, Schematic of the DEBmicroTrait model showing dynamic energy budget (DEB) allocation for a single-reserve (*E*), single-structure (*V*) heterotrophic microorganism^67^ feeding on different substrates (*S*). Diffusion-limited substrate uptake occurs through specific substrate binding sites (coloured according to substrate chemical class). Substrate uptake kinetics are described by the ECA^40^. Reserve and structural biomass are conceptualized as generalized chemical compounds characterized by macromolecular composition (C_a_H_b_O_c_N_d_) and chemical potential (*μ*_(*E*, *V*)_). Top: the coupling of catabolism and anabolism, that is, the catabolic and anabolic reactions through which energy is obtained and utilized for a metabolism in which the C source is also used as the electron donor^50^. The coupling between catabolism and anabolism exists both in reserve assimilation and structural (and extracellular enzyme) synthesis. Bottom: the sequential assimilation (*p*_A_), partitioning and dissipation of substrate and reserve compounds, with maintenance (*p*_M_) taking priority over growth (*p*_G_) and extracellular enzyme production (*p*_X_). The turnover of reserve and structure (*γ*_(*E*, *V*)_) is density-dependent^75^. Essential fluxes are labelled and defined (see also Supplementary Table 1). **b**, Workflow combining biophysical theory and genome inference to constrain DEBmicroTrait model parameters: (1) Cell size covaries with genome size^76^. (2) Codon-usage bias sets an upper bound on protein translation power (*k*_*E*_^72^). (3) The number of ribosomal RNA operons predicts translation efficiency (*y*_*VE*_^77^). (4) The cellular composition influences C supply and demand, which in turn determines the substrate binding site density required to enable substrate uptake at a rate commensurate with the maximum specific growth rate (*ρ*_porter_^84^). Binding sites can be allocated according to relative gene frequencies of transporter genes in the genome (*z*_*ρ*_). (5) Basal maintenance rate is proportional to cell volume (*k*_M_^73^). Glycoside hydrolase gene frequencies scale the constitutive extracellular enzyme production rate (*z*_X_^71^).

Here we present a genome-informed, trait-based dynamic energy budget model (DEBmicroTrait; Fig. 1 and Supplementary Table 1). This model integrates genome-predicted traits and their interactions within a dynamic environment, allowing life-history strategies and niches of soil bacteria to emerge from fundamental thermodynamic, biophysical and metabolic principles that constrain trait variation, trait linkages (defined as co-occurrence of traits in the same organism) and ultimately organism fitness. We have initially focused on integrating quantitative genomic traits that distinguish bacteria at a critical soil interface, the rhizosphere (that is, the area surrounding growing plant roots). While quantitative life-history and biophysical traits (such as genome size, maximum growth rate, cell size and rRNA operon (*rrn*) copy number) do not encompass the entirety of potential interactions and traits in bacterial ecology, they contribute substantially to explaining variations in resource utilization and provide ecological insights into species competitiveness at different stages of succession or resource depletion^20,21^. These life-history traits interact with bacterial preferences for diverse types of substrates, which can be predicted from genome sequences^22,23^. The growing development of large collections of sequenced rhizosphere bacterial genomes^22,24^, coupled with high-throughput metabolomics methods^25^, enables integration of knowledge connecting root exudate dynamics and microbial metabolism into predictive computational models of plant–microbe interactions^26^.

The rhizosphere is chemically diverse and a critical hotspot for biogeochemical transformation with high potential for C stabilization through microbial C assimilation and subsequent mineral-surface stabilization^27^. To better understand interactions between life-history traits, biophysical traits and bacterial substrate preferences, we simulated the growth of 39 soil bacteria on 82 plant exudate metabolites. We developed a theory-based approach to estimate microbial substrate uptake parameters using only genome- and substrate-derived traits. We simulated population-level estimates of key traits, including realized growth rate and C assimilation rate, across different substrate classes that are known rhizosphere exudates, and benchmarked simulations quantitatively and qualitatively against observations. We identified microbial growth strategies that arise from the multivariate trait combinations in the absence of soil matrix effects in this case. These strategies emerge from generalizable rules describing how bacterial traits interact with plant exudation traits and are defined by the cellular trade-offs associated with acquiring and assimilating diverse substrates. Most importantly, these strategies can be explained by trade-offs between growth rate (power) and growth efficiency (yield) that impose strong constraints on microbial community composition in natural habitats^28^. Furthermore, these trade-offs have implications for the formation of soil organic matter via the microbial route^29^.

## Results

**Fig. 2:**
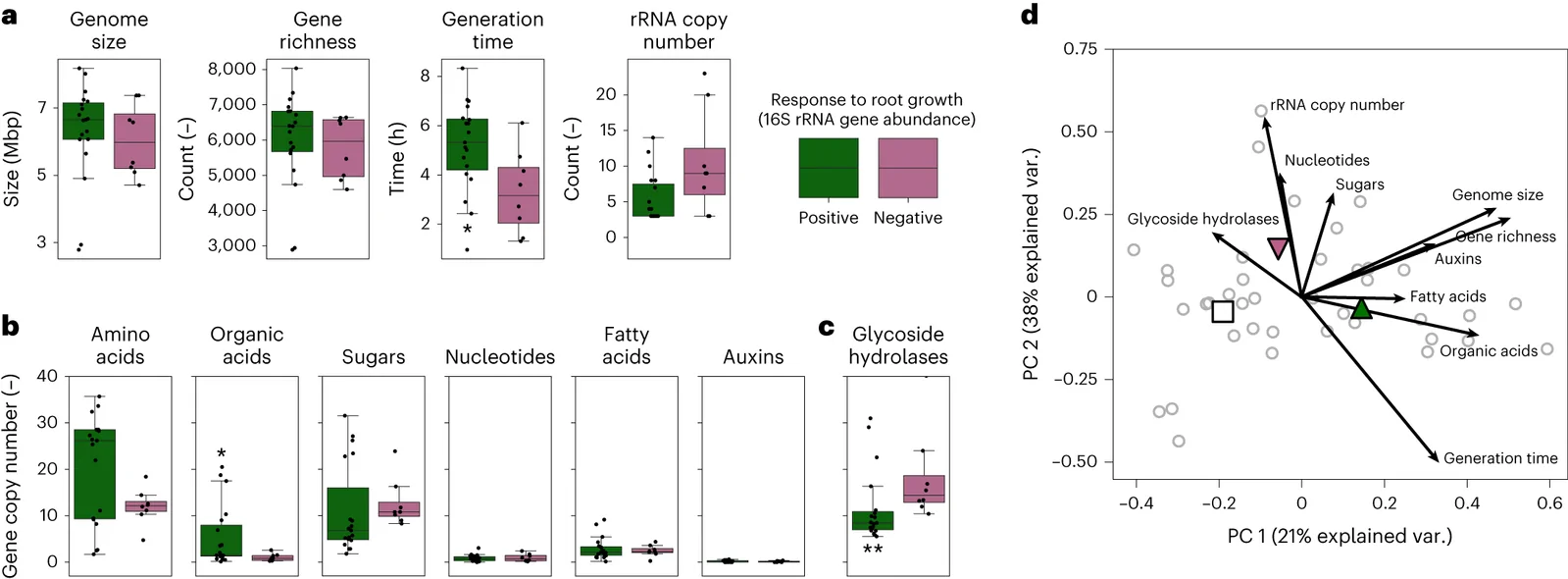
Genomic trait distributions of soil bacterial isolates classified on the basis of the response to *Avena barbata* root growth. Modified from ref. ^22^. **a**, Genome size, gene richness, minimum generation time and rRNA operon copy number predicted from genome sequences of soil isolates. **b**, Monomer transporters. **c**, Glycoside hydrolase enzymes. Gene copy numbers for monomer transporters and glycoside hydrolases were normalized by genome size. Differences in genomic trait distributions between bacteria that responded positively and negatively to root growth were evaluated using the Kruskal–Wallis one-way analysis of variance (ANOVA) (Supplementary Data 1), and traits with significant differences are annotated according to the R project standard convention^85^ used throughout the paper (***P ≤ 0.001, **P ≤ 0.01, *P ≤ 0.05). Isolate response groups were classified on the basis of changes in 16S rRNA gene abundance over the plant (*A. barbata*) developmental stages. In each boxplot, a point denotes a single isolate. The top and bottom of each box represent the 25th and 75th percentiles, the horizontal line inside each box represents the median and the whiskers represent the range of the points excluding outliers. Positive responders *n* = 19, negative responders *n* = 8. **d**, Principal component analysis illustrating covariations among genomic traits shown in **a**–**c**. Highlighted symbols represent average coordinates of positive, negative and undefined (open square) (*n* = 12) isolate response groups.

Growing roots dramatically alter the chemical and physical habitat for microorganisms and exude photosynthesis-derived C that can be broadly classified into sugars, amino acids, organic acids, fatty acids, nucleotides and auxins^30^. In fact, root exudation might be a generalizable trait with rates and composition that can be predicted from plant functional traits, with consistent temporal patterns across plant development stages^31–33^. The chemical composition of root exudates interacts with microbial substrate preferences, which are predictable from genomic traits (Fig. 2). Gene annotations assess functional potential—the capacity for organisms to perform. These soil microorganisms differ in genomic traits related to resource acquisition, particularly with regard to their metabolic potential to utilize organic acids (Fig. 2b) and their potential for plant polymer degradation using glycoside hydrolase enzymes (Fig. 2c). Contrary to expectations, we previously found that both fast-growing as well as slow-growing strains are enriched in the rhizosphere (both observed and predicted on the basis of genomic signatures^22,34,35^; Fig. 2a). The presence of potentially distinct life-history strategies challenges the long-standing assumption that living roots select for fast-growing r-strategists that compete for a small set of labile C substrates^36^. With the caveat that it is a simplification of the complex resource requirements of microorganisms, we assumed that bacteria in the rhizosphere are primarily limited by organic C inputs and energy availability. Furthermore, we hypothesized that substrate preference and substrate utilization efficiency interact to confer a growth efficiency-based fitness advantage for many bacteria in the rhizosphere. However, growth efficiency can vary widely with limiting resource concentration and the free energy content of chemical compounds that are released by plants^28^. These patterns are overlaid with physiological variations in resource use between bacteria^37–39^ that probably affect phenotypic trade-offs such as C use efficiency (CUE), suggesting that these factors combine to influence rhizosphere microbial community dynamics.

### Substrate preference in the rhizosphere

Microbial strains vary in their substrate uptake affinities, which in turn influences the composition of the root microbiome through ecological processes such as niche differentiation and competitive exclusion^25^. Recent theoretical advancements based on an analytical approximation of how diffusive substrates are intercepted by microbial cells in soil have allowed the derivation of testable relationships between substrate uptake kinetic parameters, including maximum specific reaction rates (*V*_max_) and binding half-saturation constants (also known as substrate affinity parameters, *K*), while also taking into account the biophysical, metabolic and life-history traits that influence substrate demands of soil bacteria^40^. To synthesize these relationships, we considered genomic traits that provide constraints on the interactions between cell size, cellular C density, cell surface area-to-volume ratio and growth rate potential. We used the substrate uptake potential required to support a given genome-inferred maximum specific growth rate as an objective function and investigated the variation and allometric scaling of both kinetic parameters in the equilibrium chemistry approximation (ECA) for substrate uptake^41^. The estimated substrate binding site densities (that is, transporters) were benchmarked against existing data on nutrient uptake (Supplementary Fig. 1a and Table 4) and subsequently distributed across substrate classes on the basis of the relative gene frequencies of specific transporter genes (Fig. 2b). This approach allows for the consideration of the evolutionary history of microbial substrate preference. Within each substrate class, the genome-inferred values fall within the range of variation observed in published experimental data (Supplementary Fig. 1b and Data 1). For amino acids and sugars, the differences between the values reported for cultured organisms in the literature and our predictions were statistically indistinguishable (*P* > 0.05). In contrast, for organic acids, we observed statistically significant deviations from the full data set (*P* = 0.04).

**Fig. 3:**
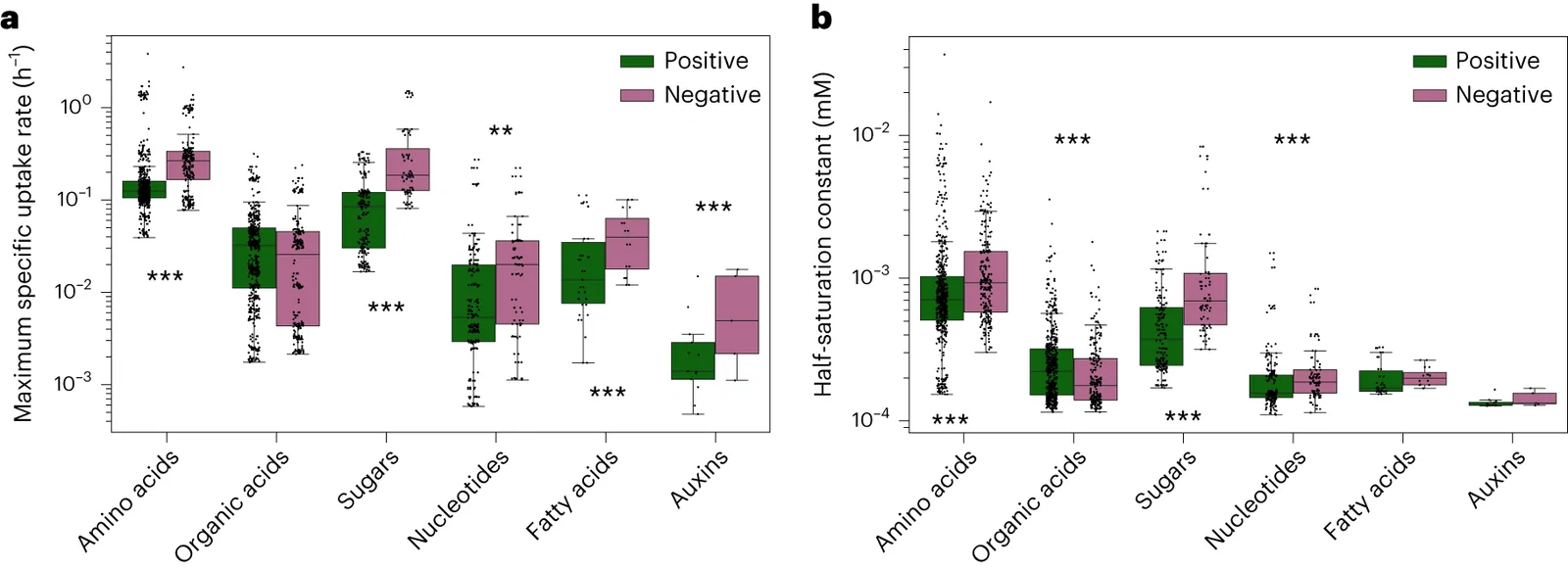
Theory-predicted distributions of root exudate metabolite uptake parameters of soil bacterial isolates. **a**, Maximum specific uptake rate. **b**, Half-saturation constant. Differences in uptake trait distributions between bacteria that responded positively or negatively to root growth were evaluated using the Kruskal–Wallis one-way ANOVA (Supplementary Data 1). In each boxplot, a point denotes a single substrate–consumer relation in the ECA^40^. The top and bottom of each box represent the 25th and 75th percentiles, the horizontal line inside each box represents the median and the whiskers represent the range of the points excluding outliers. Substrates *n* = 82, consumers *n* = 27.

**Extended Data Fig. 1:**
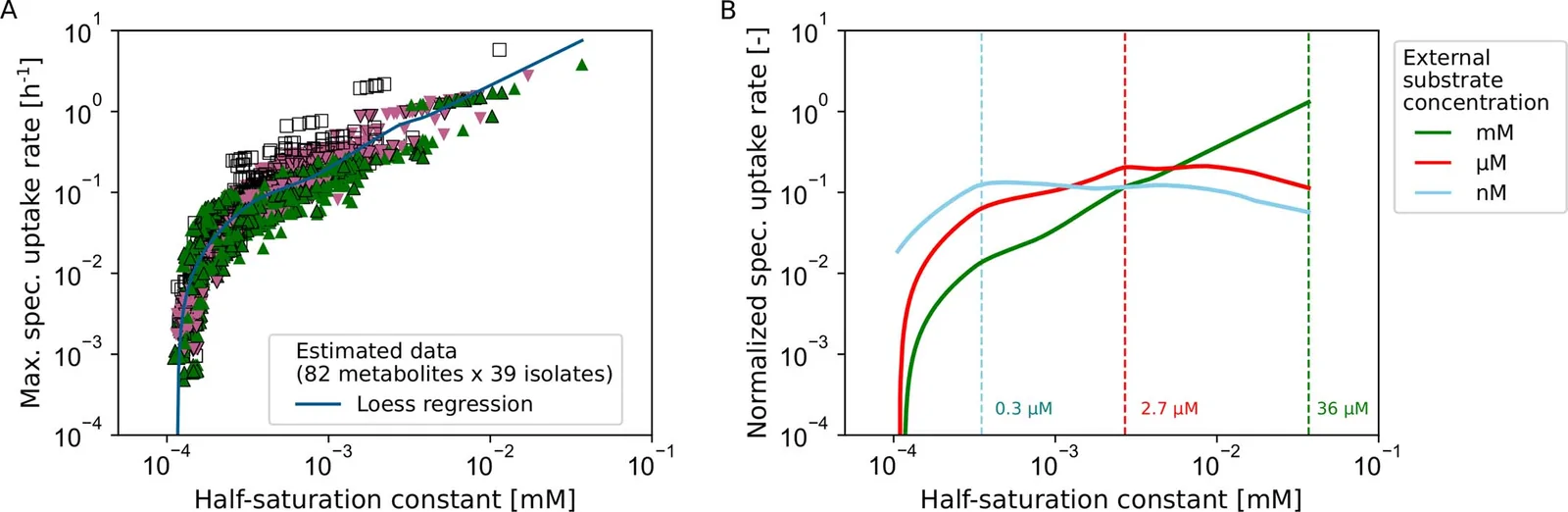
Trade-offs between substrate uptake parameters and optimal affinity parameter values at different external substrate concentrations. **A** Trade-off curve between predicted maximum specific uptake rate (${{{{\rm{V}}}}}_{\max }$) and half-saturation constant (also known as substrate affinity parameter, K) in the equilibrium chemistry approximation (ECA^40^) for substrate uptake across substrates (n = 82) and isolates (n = 39). Isolates are colored by their response to root growth (green: positive, magenta: negative, white: undefined). **B** Normalized specific substrate uptake rate (J) as a function of the affinity parameter (K) for three different external substrate concentrations (D). The dashed lines indicate optimal affinity parameter values (K^opt^). In **A** and **B**, the solid lines correspond to locally weighted linear regression models across substrates and isolates (n = 3198).

The predicted uptake kinetic parameters vary widely due to factors such as substrate diffusivity, cell size and biomass-specific accessible substrate binding sites in the rhizosphere (Fig. 3). Soil bacteria previously defined as responding negatively to root growth^22^ may actually achieve significantly higher maximum specific uptake rates to match their higher genome-predicted maximum specific growth rates (Fig. 3a). However, their reduced ability to uptake organic acids and auxins that can be prominent in root exudates (Fig. 2b) outweighs their higher maximum growth potential (Fig. 2a). Overall, the optimization of *V*_max_ and *K* modulates the competitiveness of rhizosphere organisms according to the placement of their uptake strategies across a concave trade-off curve (Extended Data Fig. 1a). This concave shape allows for the maintenance of optimal substrate uptake phenotypes, enabling organisms to thrive under varying external substrate concentrations^42^. Amino acids and sugars are generally considered to be the most abundant classes in terms of the total amount exuded^43^. Our analysis predicts that the affinity constants of these rhizosphere bacteria for amino acids are nearly optimal at micromolar concentrations (Extended Data Fig. 1b). In addition, we predict that increasing substrate affinity by increasing the substrate binding site density beyond ~0.1% of the cell surface area provides little benefit to soil microorganisms (Supplementary Fig. 2b). This finding contrasts with well-mixed systems at equilibrium, such as oligotrophic marine systems, where the activity of loaded binding proteins monotonically increases affinity and specific uptake rate at low substrate concentrations^44^. It highlights that the ecological trade-offs that define fitness in soil may differ from those that define life-history dichotomies in other habitats^45^. We observe that the substrate affinity constant that maximizes the specific uptake rate at low concentrations is closest to the average value for organic acids (0.26 μM; Extended Data Fig. 1b), suggesting that certain soil bacteria have evolved specialized affinity uptake systems to colonize specific metabolic niches in soil.

**Fig. 4:**
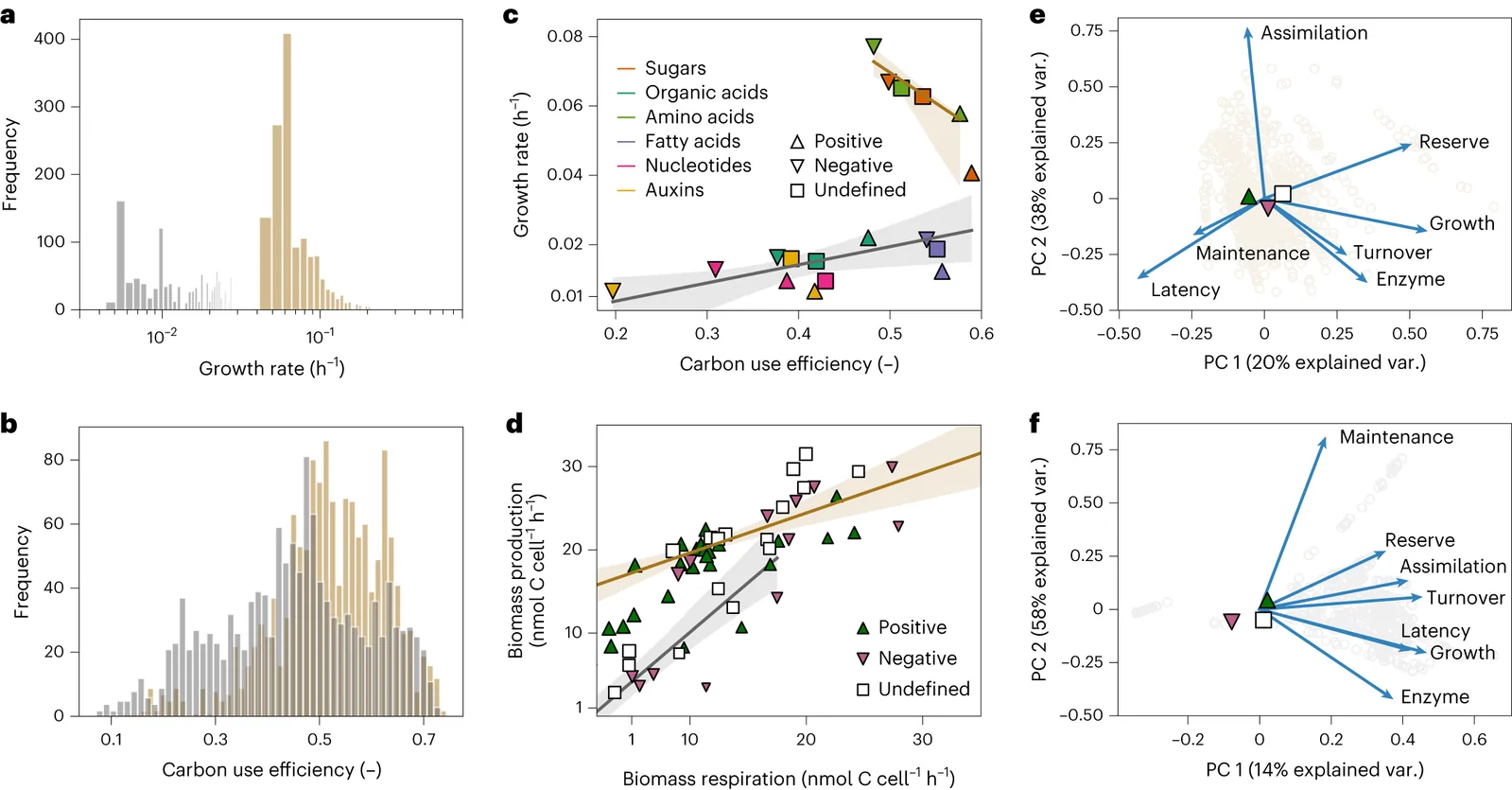
Phenotypic traits and trade-offs during batch growth on root exudate metabolites. **a**,**b**, Histogram of predicted growth rate (**a**) and predicted CUE (**b**) across isolates and root exudate metabolites in DEBmicroTrait batch simulations. Throughout plots **a**–**f**, two distinct growth regimes are distinguished by brown (high growth regime) and grey (low growth regime) colours on the basis of the bimodal growth rate distribution shown in **a**. **c**, Relationships between realized growth rate and carbon use efficiency. Median trait values are plotted using different colours and shape depending on substrate class (sugars, organic acids, amino acids, fatty acids, nucleotides, auxins) and isolate response to plant root growth (positive, negative, undefined). **d**, Relationships between biomass production and respiration rates. Symbol size is scaled by carbon use efficiency. In **c** and **d**, solid lines indicate the observed regression lines, while shaded areas indicate the 95% confidence bands. **e**,**f**, Principal component analysis illustrating covariations among modelled fluxes delineating growth strategies of isolates at high (**e**) and low (**f**) realized growth rates. Highlighted symbols represent average coordinates of positive, negative and undefined isolate response groups. The significance threshold for two-sided *P* values was set at 0.05. The principal components of the different response groups are significantly different (*H*_PC1_(2) = 329, *H*_PC2_(2) = 105, *P* = 2.2 × 10^−16^). Dunn’s test with Benjamini–Hochberg correction confirmed the significant differences between positive and negative responders along PC1 (PC2) (*P* = 2 × 10^−16^ (9.3 × 10^−4^)), and negative and undefined responders (*P* = 1.6 × 10^−^^5^ (2 × 10^−16^)) in the high growth regime. In the low growth regime, the ecological strategies of negative and undefined responders coalesce along both principal components (*P* = 0.43 (0.09)). They differ significantly from positive responders (*P* = 2.5 × 10^−5^). Substrates *n* = 82, consumers *n* = 39, simulations *n* = 3198.

However, resource acquisition traits are only one facet of ecological strategy variation, and interactions with cellular resource allocation strategies are key. To address this, we analysed biomass production (BP) and respiration (BR) rates of 39 soil bacteria growing on 82 root metabolites across combined 3,198 batch simulations (Fig. 4) to explore relationships between realized growth rate and yield (that is, CUE), calculated as BP/(BP+BR).

### Power–yield signatures in bacterial rhizosphere succession

Microbial access to soil C, as well as species-specific differences in the energetic demands required to use different substrate classes, select for distinct growth regimes in the rhizosphere (Fig. 4a). While growth rates in the low growth rate regime (0.0044–0.039 h^–1^) are reflective of growth rates of autochthonous bacteria occurring in unamended soils^46^, the growth rates in the high growth rate regime span typical values observed in pure cultures under laboratory conditions (0.039–0.46 h^−1^). Maximum realized growth rates were partially confirmed with measured growth rates at C concentrations representative of the original growth media used to cultivate the bacterial isolates (*r*^2^ = 0.85; Supplementary Fig. 3 and Table 5). Potential CUE values range from 0.07 to 0.74 with a median of 0.49, suggesting that on average approximately half of the consumed C is typically lost via respiration (Fig. 4b). Bacterial traits related to the assimilation of C had the largest influence on model-simulated CUE (Supplementary Fig. 4). A similar amount of variation in CUE was explained by phylogeny at class level (20%) relative to substrate class (15%; Supplementary Table 2), indicating that interactions between substrate traits (for example, mean differences in molecular size and nominal oxidation state of C among substrate classes) and microbial traits (for example, transporter gene frequencies, protein synthesis efficiency, relative maintenance costs) provide a strong foundation for this aspect of soil bacterial ecology^47,48^.

Bacterial growth rate and CUE trade off during growth on substrates of high bioavailability (defined by solubility and hydrophobicity^49^; Supplementary Data 1) exuded early during plant growth (sugars, amino acids), highlighting an early successional growth strategy where power is optimized over yield (*F*_1,3_ = 13.83, *r*^2^ = 0.82, *P* = 0.03; Fig. 4c). Maximum CUE is achieved for those bacteria that responded positively to root growth when simulated with glucose as the substrate, while maximum growth rates occur at suboptimal CUE for organisms with the fastest growth rates. The rate–yield trade-off emerges from thermodynamic constraints reflecting energy generation and transfer from catabolism to anabolism, resulting in decreasing structural biomass yield to accommodate increasing protein synthesis rates (Supplementary Fig. 5), as well as increased maintenance costs at high growth rates. Energy dissipation increases with the rate of C uptake^50^, resulting in lower thermodynamic efficiency for growth on amino acids as compared with sugars, and an overall disproportional scaling between biomass production and respiration rates (with slope that is significantly different from one, *β* ∈ (0.56, 0.63), *t*_1,437_ = −24, *P* < 1 × 10^−5^; Fig. 4d). Fast growth is constrained by internal substrate limitation caused by the accelerated dilution of storage compounds due to (volume) growth, indicating that these bacteria grow faster than they can assimilate new substrate during early rhizosphere successional growth stages. The corresponding realized growth rates are strongly correlated with the number of *rrn* copies in the genome (Supplementary Table 3), where this single genomic trait explains about a third of the variation (*r*^2^ = 0.30). This supports the hypothesis that genomic traits related to maximum growth potential are good predictors of growth responses to initial resource pulses in the rhizosphere^46^.

**Extended Data Fig. 2:**
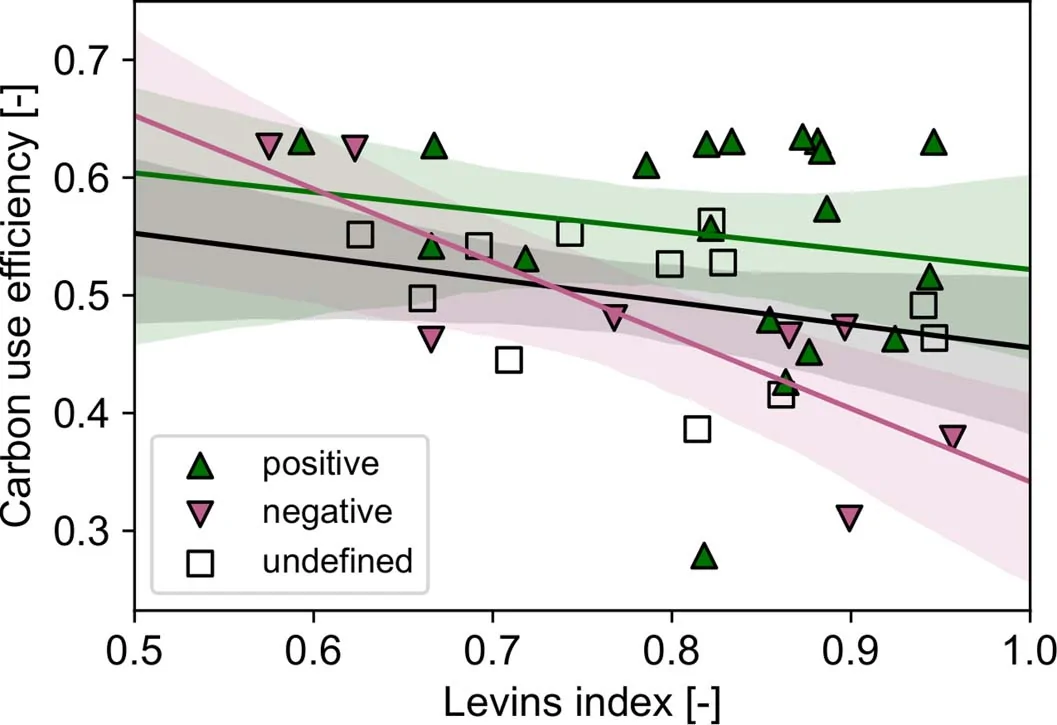
Niche breadth of isolates for growth on mixed root exudate metabolites. Relationship between carbon use efficiency and resource niche breadth (Levins index^83^) of isolates for growth in a mixed medium composed of 82 root exudate metabolites. Isolates are colored by their response to root growth (green: positive, magenta: negative, white: undefined). The solid lines indicate the respective regression line (positive: F_1,17_ = 0.44, r^2^ = 0.03, p = 0.52, negative: F_1,6_ = 10.99, r^2^ = 0.65, p = 0.01, undefined: F_1,10_ = 1.31, r^2^ = 0.12, p = 0.28), while shaded areas indicate the 95% confidence bands. Niche breadth was unimodally distributed across isolates based on Hartigan’s dip test (all: D = 0.04, p = 0.76, positive: D = 0.07, p = 0.68, negative: D = 0.11, p = 0.52, undefined: D = 0.08, p = 0.81).

The contribution of bacteria to soil C cycling in the rhizosphere is primarily determined by anabolic processes^51^ that control the allocation of C and energy for maintenance, the synthesis of storage compounds, as well as extracellular enzyme production and assimilation. These processes represent nearly independent (orthogonal) axes of variation (Fig. 4e), providing compelling evidence for the ecological classification of rhizosphere bacteria into the yield–acquisition–stress (Y–A–S) framework of life-history strategies^5^. Within this framework, bacteria that responded positively to root growth could be considered Y-strategists, actively exploiting available low-molecular-weight C sources through direct capture and assimilation. However, these bacteria also showed greater latency in their growth response (Kruskall–Wallis test, *P* < 1 × 10^−16^), suggesting that they may be assimilating additional C in the rhizosphere that has been transformed by previous microbial processing. Interestingly, the absence of a trade-off between niche breadth and yield (Extended Data Fig. 2) indicates a high degree of resource specialization among these organisms. Niche differentiation is further facilitated by resource acquisition strategies of negative responders (A-strategists), which appear to be better adapted to the breakdown of root polymeric carbohydrates via the constitutive expression of carbohydrate-active enzymes^52^. Both Y- and A-strategies align with functional differences encoded in genomes along the copiotroph–oligotroph continuum. Copiotrophs frequently possess a greater prevalence of gene families involved in transcription, transport and metabolism of carbohydrates and amino acids, as well as carbohydrate-active enzymes such as glycoside hydrolases and polysaccharide lyases. This overall strategy enables copiotrophs to rapidly acquire nutrients and produce proteins^35^.

Across time, we find that yield and resource acquisition strategies are tightly linked along gradients of resource availability, as bacteria grow more slowly on root exudates that are released during later plant developmental stages (organic acids, fatty acids, nucleosides, auxins). For slower-growing bacteria, only 4% of the variance in growth rates is explained by *rrn* copy number and genome size (Supplementary Table 3), consistent with findings that there is essentially no selective advantage to optimizing translational power via codon usage at low growth rates^35^. The observed proportional scaling between biomass production and respiration (with slope *β* ∈ (0.95, 1.05), *t*_1,332_ = 0.06, *P* = 0.95; Fig. 4d) is consistent with an overall oligotrophic strategy of energy production, conversion and cell maintenance. Indeed, basal maintenance requirements and extracellular enzyme production explain most of the variability in the growth strategies of isolates at low growth rates (Fig. 4f). The ecological strategies of bacteria that responded negatively to root growth or showed no response to root growth coalesced along both principal components, implying that succession in the rhizosphere is accompanied by a significant decrease in functional diversity, which is consistent with previous observations^53^. As the plant matures, the soil surrounding roots harbours more conservative, slower-growing organisms that do not exhibit a trade-off between growth rate and efficiency since turnover is slower for these organisms (*F*_1,11_ = 7.74, *r*^2^ = 0.41, *P* = 0.02; Fig. 4c). Taken together with CUE values (0.07–0.74) that span almost the whole range of values typically observed in soil (Fig. 4a)^54^, we hypothesized that selection for efficiency via substrate preference is a primary driver of rhizosphere community composition during the later stages of plant development.

### Rhizosphere carbon stabilization via resource specialization

**Fig. 5:**
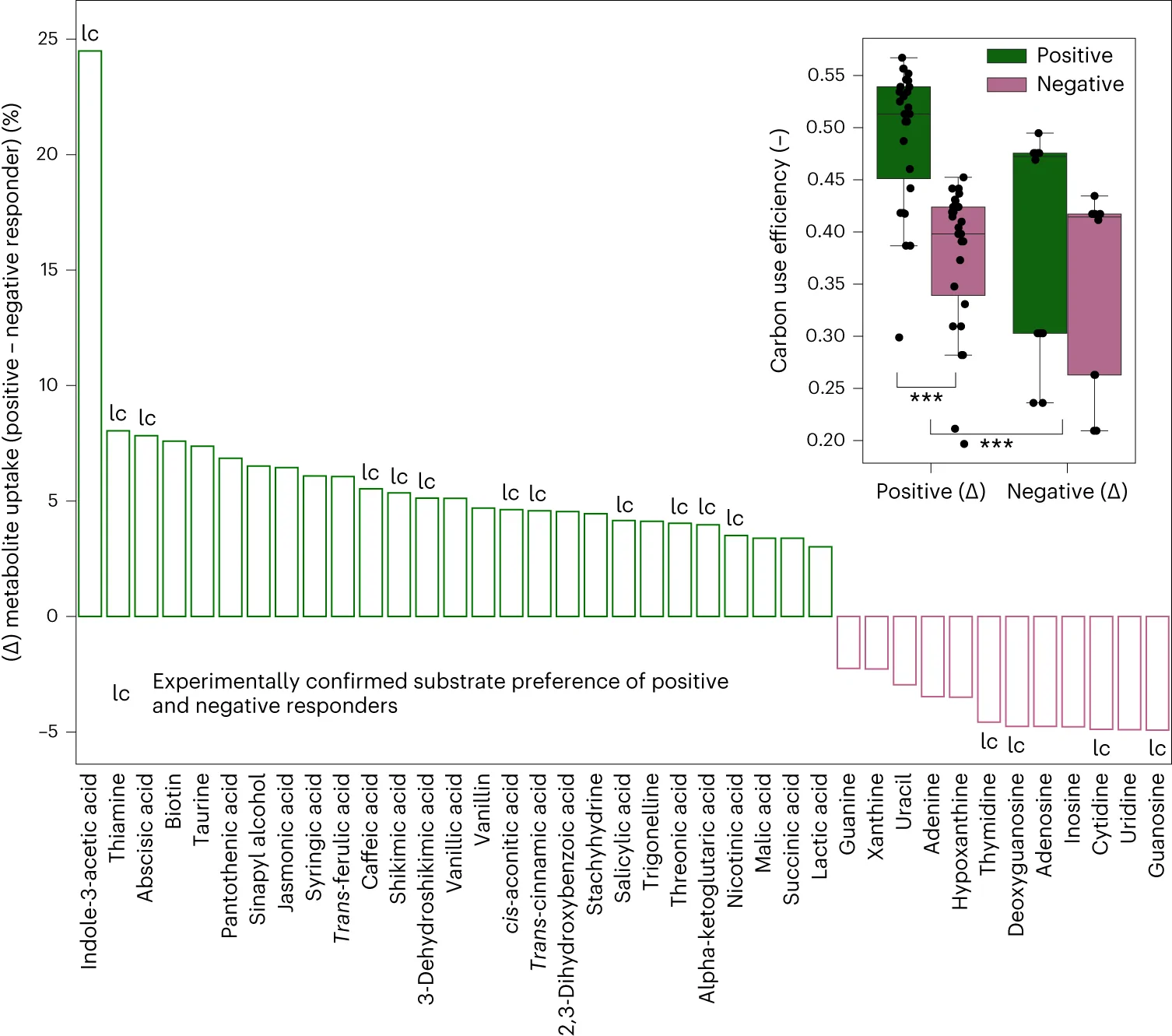
Substrate preference and carbon use efficiency in mixed root exudate medium. Substrates with the largest differences in uptake are shown on the *x* axis (*n* = 39). Each bar corresponds to differences in substrate uptake quantified as a percentage of the difference in substrate depletion from the medium. Substrate uptake preferences that were confirmed experimentally^22^ are denoted by the letters ‘lc’. Inset: differences in carbon use efficiency for substrates that were preferentially consumed by bacteria that responded positively (positive (Δ), *n* = 27) and negatively (negative (Δ), *n* = 12) to root growth. Differences in carbon use efficiency within (*χ*^2^ = 9.6, *P* = 0.002) and between (*χ*^2^ = 23.6, *P* = 1 × 10^−6^) substrate preference groups were evaluated using the Kruskal–Wallis one-way ANOVA. In each boxplot, a point denotes a single substrate–isolate pair. The top and bottom of each box represent the 25th and 75th percentiles, the horizontal line inside each box represents the median and the whiskers represent the range of the points excluding outliers. Substrates *n* = 82, consumers *n* = 27, simulations *n* = 27.

To test whether bacterial preference for specific substrates could interact with CUE to confer a selective advantage in the rhizosphere, we analysed simulations of growth on mixed media (a combination of 82 exudate substrates) for differences in substrate uptake across the positive and negative rhizosphere responder groups. We found that 39 of the 82 exudate substrates show substantial differences in substrate uptake between bacteria that responded positively or negatively to root growth (Fig. 5). For 16 of the 39 root exudates that had previously been identified experimentally, the largest cumulative differences in substrate uptake by bacteria that responded positively to root growth were for plant hormones (indole-3 acetic acid, abscisic acid), followed by a cluster of aromatic organic acids (caffeic, shikimic, 3-dehydroshikimic, *trans*-cinnamic, salicylic, nicotinic). Nucleosides, on the other hand, were more preferentially consumed by the bacteria that responded negatively to root growth. Differences in uptake of 16 out of the 39 substrates agree qualitatively with uptake from the growth medium measured by liquid chromatography–mass spectrometry (LC–MS), without accounting for interactions with the soil matrix^22^. Furthermore, bacteria that responded positively to root growth had a 39% higher growth efficiency on average for organic acids with aromatic rings than those isolates that responded negatively to growing roots (Fig. 5 inset).

## Discussion

Root–microbial interactions have significant effects on the soil C cycle, altering the amount and types of organic matter that become associated with mineral surfaces. Focusing on recent studies that have identified direct predictive links between plant exudate composition and rhizosphere community assembly, and a theory-based approach to predict microbial substrate uptake kinetic traits directly from genome sequences, we synthesized a suite of genome-derived traits into model-based predictions of life-history strategies for a set of soil bacteria. We found that interacting microbial traits (maximum specific growth rate, substrate uptake kinetics, ribosome biosynthesis potential and extracellular enzyme synthesis) have additional interactions with the dynamics of root exudate chemistry, creating emergent patterns of bacterial C use efficiency. These combinations of traits manifest as life-history strategies and have consequences for the path that small molecules take on the way to becoming stabilized soil organic matter.

**Fig. 6:**
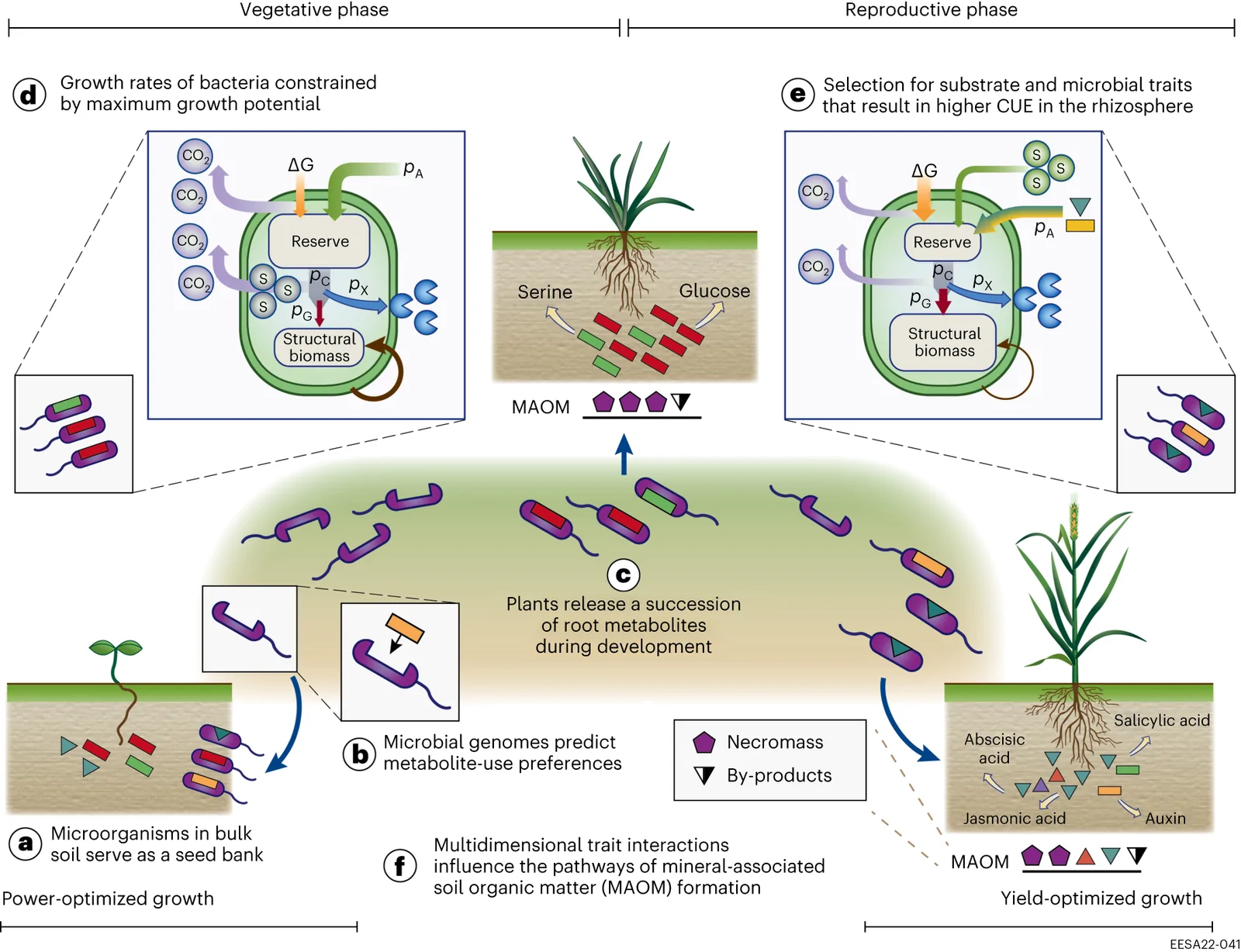
Bacterial and root exudate metabolite traits interact to determine growth strategies in the rhizosphere. **a**,**b**, The microorganisms in bulk soil function as a seed bank and vary in their genetic potential to import and utilize distinct metabolites as substrates. **c**, Plants are genetically programmed to release a succession of root exudate metabolites throughout developmental stages (shown here are the vegetative and reproductive phases, with distinct metabolites coloured according to the six chemical classes used throughout the paper). **d**, During the vegetative growth phase, bacteria grow faster than they can assimilate new substrate. Rapid growth on sugars and amino acids is constrained by maximum growth potential and correlated with high biomass turnover. **e**, Bacteria grow more slowly on root exudates that are released during the reproductive growth phase (organic acids, fatty acids, auxins). Substrate limitation occurs from environmental supply, except for select root metabolites that are preferentially consumed in the rhizosphere. These compounds select for organisms with traits (higher relative transporter gene frequencies, lower relative maintenance investment, higher protein synthesis efficiency, lower biomass turnover) that result in higher carbon use efficiency than typically observed experimentally when phenolic or polyvalent organic acids are added to bulk soil^55^. These results suggest that microbial community assembly patterns can be predicted from models coupling substrate-use preferences and chemical succession in the rhizosphere^22,86^. **f**, Understanding these patterns can guide strategies for engineering plant phenotypes and microbial communities, enhancing carbon stabilization through microbial carbon assimilation and subsequent stabilization on mineral surfaces.

Root exudates can follow distinct pathways before integrating into the soil matrix: direct sorption to mineral surfaces or microbial transformation^55^. While root-derived compounds may rapidly exchange with mineral surfaces^56^, it is now widely recognized that the direct microbial transformation of labile photosynthate C into stabilized forms allows microorganisms to contribute disproportionately to persistent C in the soil^29,57,58^. On the basis of our results, we propose that multidimensional trait interactions influence the pathways of mineral-associated soil organic matter (SOM) formation throughout the plant growing season (Fig. 6). As microbial composition and abundance change over the growing season, the initial exudation of sugars and amino acids with weak sorption to mineral surfaces coincides with power-optimized growth strategies (resulting in more C overall into microbes). C inputs from sugars and amino acids stimulate the growth of resource acquisition (A-) strategists and may accelerate the decomposition of surrounding organic residues, mostly of plant origin, as previously observed in this study system^52,59^. The growth response of individuals is highly dynamic, with fast growth correlating with high density-dependent turnover of microbial biomass (Fig. 4e). This rapid initial turnover of biomass during the vegetative phase implies that secondary processing, fuelled by the products of microbial anabolism, comprises an important component of the C that cycles through rhizosphere communities^60^. While the metabolic products of microbes subsisting on glucose or amino acids earlier in the growing season may contribute to direct mineral-surface stabilization^61^, the subsequent emergence of more yield-optimized guilds (more C per specific microbe) may selectively enhance the mineral stabilization of compounds. This enhancement occurs through the deposition of senesced microbial biomass containing greater proportions of lipids and proteins and fewer aromatics^62^. Therefore, these pathways may represent two distinct routes to mineral stabilization of C—which of these pathways is quantitatively most effective in creating mineral-associated SOM remains to be determined.

Using batch simulations to infer microbial life-history strategies and niches entails a trade-off. This is because the behaviour of organisms in culture may not always align with their performance in natural settings^46^. However, ground truthing of genome-inferred microbial traits is needed to enhance the credibility and confidence in genome-informed model predictions. Furthermore, using a consistent methodology to measure CUE across a broad range of microbial taxa is necessary to determine how physiological variation in substrate use between rhizosphere bacteria impacts CUE^37,38^. The same holds true for substrate uptake measurements, the accuracy of which is significantly influenced by experimental design elements, such as incubation duration and the preceding physiological state of the organisms. Consequently, deviations from model predictions might potentially stem from estimation inaccuracies or a lack of correlation between the half-saturation concentration for uptake and the half-saturation concentration for growth over extended incubation periods^63^. The simulation results presented here represent model-based hypotheses to confirm with appropriately designed experiments. These include considering a range of abiotic and biotic factors such as diurnal exudation dynamics^64^, competition with roots and mineral surfaces for essential nutrients^65^, and interactions with other organisms^66^. By integrating across a hierarchy of traits, genome-informed trait-based modelling facilitates the generation and testing of hypotheses and can provide a robust foundation for the data-driven representation of microorganisms in many complex systems.

## Methods

### DEBmicroTrait model description

DEBmicroTrait assumes that microbial metabolism can be described by the standard dynamic energy budget (DEB) theory^67^. DEB theory partitions the total biomass into generalized chemical compounds (reserve and structural biomass) with specific functions: (1) reserve, denoted *E*, comprising the cellular growth machinery, including ribosomal proteins and RNA involved in the biosynthesis of proteins and/or additional ribosomes, as well as localized storage compounds (for example, glycogen, polyhydroxybutyrate) that buffer metabolism against external fluctuations in resource supply and (2) structural biomass, denoted *V*, comprising essential cellular proteins, DNA and other macromolecules that make up the cell wall and membrane^68^. Structural biomass is subjected to maintenance while reserves are subjected to continuous external supply and utilization (Fig. 1). The utilization of reserves follows first-order dynamics when expressed as a reserve density, *m*_*E*_ = *E*/*V*. The first-order turnover rate is given by

1 $${k}_{E}=v/{L}_{c}={p}_{Am}/{m}_{E}^{* },$$

where *v* is an energy conductance parameter, ${L}_{\rm{c}}={V}_{\rm{c}}^{1/3}$ is volumetric length, *p*_Am_ is the maximum assimilation flux and ${m}_{E}^{* }$ represents the ratio of assimilation and mobilization fluxes, that is, the reserve capacity of the organism that is reached at long exposure to high substrate concentrations. The reserve pool buffers between environmental substrate uptake and microbial cell metabolism^69^.

If the net synthesis rates of reserve and structure are balanced, weak homoeostasis implies that substrate limitation may occur inside the cell, capturing the dilution of reserve compounds due to (volume-) growth at a rate proportional to the reserve density^70^. It follows that the reserve mobilization power of the organism is given by the difference between the first-order turnover rate of reserve and dilution by growth,

2 $${p}_{\rm{C}}={\mu }_{E}({k}_{E}{m}_{E}-{m}_{E}r),$$

where *μ*_*E*_, measured in Gibbs energy per mol or C-mol, is the chemical potential of reserve and $r\equiv \frac{1}{V}\frac{dV}{dt}$ denotes the specific growth rate of the organism.

The reserve mobilization power, *p*_C_, is partitioned between growth, *p*_G_, maintenance, *p*_M_, and extracellular enzyme production, *p*_X_, power, that is,

3 $${p}_{\rm{C}}={p}_{\rm{G}}+{p}_{\rm{M}}+{p}_{\rm{X}},$$

where, in the absence of regulation, extracellular enzymes are produced constitutively at a rate proportional to the specific growth rate^71^. Dissipative loss in the growth and protein synthesis machinery is linked to the energetic cost of functional protein increasing with translation speed^72^. The basal maintenance rate is taken as proportional to cell volume—a valid assumption if the cumulative cost of protein turnover and replacement plays an important role in the overall energy budget and the amount of protein is proportional to cell volume^73^. Since the maintenance costs of protein turnover are preferentially on the expense of reserve materials, selection for high translation power (leading to reduced translational yield, *y*_*VE*_) may also trade-off with the tolerance for starvation of the organism. However, somatic maintenance costs, *k*_M_, can be supplemented with structural biomass when starvation leads to the depletion of the reserve density, albeit at higher overhead costs in the transformation^74^.

An explicit equation for the specific growth rate is obtained by combining equations (1) and (2) as

4 $$r=\frac{{p}_{\rm{G}}}{{\mu }_{E}{y}_{EV}}=\frac{{k}_{E}{m}_{E}-({p}_{\rm{M}}+{p}_{\rm{X}})/{\mu }_{E}}{{m}_{E}+{y}_{EV}}$$

where *y*_*EV*_ = 1/*y*_*VE*_ denotes the inverse of the structural growth efficiency. The inefficiencies associated with assimilation, growth, maintenance and enzyme production are captured as respiratory CO_2_ losses (BR) and contribute to the overall CUE of the organism. CUE was calculated as BP/(BP+BR), where BP denotes total reserve and structural biomass production taking into account density-dependent biomass turnover^75^.

A schematic for the coupling of anabolism and catabolism, as well as C and energy allocation in DEBmicroTrait is shown in Fig. 1. DEBmicroTrait trait integration and auxiliary equations to translate traits into model quantities are described below. An overview of the trait integration framework is provided in Supplementary Table 1.

#### Model assumptions for ribosome requirements

In our choice of parameterization, we assumed that natural selection has fine-tuned the translational power of ribosomes for optimal activity in a given environment^68^. Shifts in translational power across different bacteria follow allometric trends in macromolecular composition according to

5 $${k}_{E}=\frac{{r}_{\rm{max}}{V}_{\rm{P}}}{{V}_{\rm{R}}},$$

where *r*_max_ is the genome-inferred maximum specific growth rate, *V*_P_ is the total protein volume and *V*_R_ is the ribosomal volume of the cell. *V*_p_ scales sublinearly with total cellular volume and impacts the ribosomal volume by considering how many ribosomes are required to replicate all ribosomes and proteins within a division cycle while also replacing proteins and ribosomes that have been degraded^76^. This places a lower bound on overall ribosomal volume of the cell according to the following inequality,

6 $${V}_{\rm{R}}\ge \frac{{\bar{l}}_{\rm{P}}{v}_{\rm{P}}{N}_{\rm{P}}(\phi /r+1)}{{v}_{\rm{R}}({\bar{r}}_{\rm{R}}/r-{\bar{l}}_{\rm{R}}(\eta /r+1))},$$

where *r* is growth rate, ${\bar{l}}_{\rm{R}}$ is the average length of a ribosome in base pairs, ${\bar{r}}_{\rm{R}}$ is the maximum base pair processing rate of the ribosome, ${\bar{l}}_{\rm{P}}$ is the average protein length found to be invariant across bacteria, *η* and *ϕ* are specific degradation rates for ribosomes and proteins, *N*_P_ is the total number of proteins, and ${\bar{v}}_{\rm{P}}$ and ${\bar{v}}_{\rm{R}}$ represent the volume of an average protein or ribosome, respectively. While conceptually simple, the model explains more variance in translation phenotypes across species (*r*^2^ = 0.78, *n* = 11; Supplementary Fig. 6a) than genomic signatures based on *rrn* copy number alone (*r*^2^ = 0.49; Supplementary Fig. 6b). For simplicity, we assumed that *rrn* copy number is a good predictor of translational yield^77^. Consequently, we found that the fraction of reserve that is mobilized for growth, that is, *y*_*V*_ = *r*/(*k*_*E*_*m*_*E*_), is negatively correlated with *k*_*E*_ at high *k*_*E*_ (Supplementary Fig. 5). At low *k*_*E*_, the structural biomass yield remains relatively constant because reserves can be mobilized efficiently. As a result, maximum growth rates can occur at suboptimal yield^78^.

#### Membrane requirements for substrate uptake

ECA kinetics provide a robust mathematical representation of complex substrate–consumer interactions in soil environments^41^. When applied to diffusive substrate uptake, the substrate affinity *K*_*ij*_ (mM) for the binding of substrate *D*_*i*_ (*i* ∈ [1, ... , *I*]) to free cellular binding sites *B*_*j*_ (*j* ∈ [1, ... , *J*]) in chemical equilibrium can be approximated as

7 $${K}_{ij}={K}_{0,ij}\left(1+\frac{{k}_{ij}^{+}{B}_{j,\rm{T}}}{4\pi {\tilde{D}}_{i}{r}_{{\rm{c}},j}{n}_{j}}\right),$$

where ${K}_{0,ij} \sim {k}_{2,ij}/{k}_{ij}^{+}$ is the ratio of forward reaction coefficients, *B*_*j*,T_ denotes total (free and occupied) binding sites, ${\tilde{D}}_{i}$ is the substrate diffusivity (m^2^ s^−1^), *r*_c,*j*_ is the spherical cell radius (m) and *n*_*j*_ is the cell number density (m^−3^). The maximum substrate processing rate *k*_2,*ij*_ (s^−1^) defines the maximum specific uptake rate according to *V*_max,*ij*_ = *k*_2,*ij*_*B*_*j*,T_. Hence, if a cell increases its volumetric binding site density (*B*_*j*,T_/*n*_*j*_), it decreases its substrate affinity. At the same time, decreasing the volumetric size while keeping the same area-specific binding site density, *B*_*j*,T_ = *n*_*j*_*ρ*_*B*,*j*_4*πr*_*c*,*j*_, can increase substrate affinity.

We considered binding site densities in the context of cellular substrate supply and demand under conditions of balanced growth. Then, the ratios of extensive properties (that is, intensive properties, such as the reserve density) remain constant^79^. It follows that the reserve density is given by the ratio of assimilation power to reserve turnover rate (equation 1). Furthermore, cells have evolved optimal protein densities in cellular compartments (for example, the cell membrane) that maximize reaction rates^80^. Cellular demand depends on biochemical processes associated with substrate assimilation into generalized reserve compounds. Accordingly, cells can differ in their assimilation yield as given by

8 $${y}_{ED}=\frac{1}{{Y}_{\rm{cat}}^{\rm{c}}+\lambda {Y}_{\rm{an}}^{\rm{c}}},\lambda =\frac{\Delta {G}_{\rm{an}}+\Delta {G}_{\rm{diss}}}{-\Delta {G}_{\rm{cat}}},$$

where *λ* couples the stoichiometric vectors for catabolism (${Y}_{\rm{cat}}^{\;\rm{c}}$) and anabolism (${Y}_{\rm{an}}^{\;\rm{c}}$) by determining how many times the catabolic reaction (the energy production through substrate degradation, Δ*G*_cat_) needs to run to provide the Gibbs energy for anabolism (Δ*G*_an_), with the remaining energy dissipated into the environment (Δ*G*_diss_). Determination of *λ* requires the calculation of the Gibbs free energy changes Δ*G*_cat_ and Δ*G*_an_, the latter of which is equivalent to the energy conversion to generalized reserve compounds, plus the synthesis costs of membrane-bound binding site proteins, that is, $\Delta {G}_{\rm{an}}=\Delta {G}_{\rm{block}}+\Delta {G}_{{\rho }_{\rm{porter}}}$ in our formulation. For simplicity, the elemental composition of reserve was treated as similar to structural cell components and follows allometric trends in cellular biomolecule composition with volume^76^. The Gibbs energy of dissipation denotes the Gibbs energy change of the conversion of the biomass building block to the different biomass components, that is, Δ*G*_diss_ = Δ*G*_syn_/*ν*, where *ν* is a constant fraction of the energy dissipated in all enzymatic steps of the process. We assumed that Δ*G*_syn_ is constant^81^, but organisms can differ in their protein synthesis efficiency as described above.

The cellular membrane area that needs to be covered with binding sites (*ρ*_porter_) to reach transport rates commensurate with maximum specific growth rates was obtained by solving equations (1), (4) and (8). The estimated binding site densities of isolates matched for specific substrate molecules in the rhizosphere ranged from 0.0005% to 0.19%, with a median covering ~0.1% of the cell surface. The average estimated binding site density for cumulative uptake of plant metabolites amounted to ~0.08, or 8% of the total membrane area. These estimates were then scaled using normalized relative gene frequencies of specific transporter genes in the genome of isolates. To normalize the results per unit C microbial biomass^40^, we calculated the number of cells in one mol C equivalent biomass (*λ*_B_) using the allometric scaling of cell dry mass components (*M*_dry_) and the assumption that 47% of dry biomass is C^76^.

To represent substrate competition in a network of single-substrate to product reactions, the consumption of substrate *D*_*i*_ by a consumer *B*_*j*_ is then given by

9 $${j}_{D,ij}=\frac{d{D}_{i,\;j}}{dt}=-{k}_{2,ij}^{+}{N}_{ij}{B}_{j}\left(\frac{{F}_{{\rm{c}},\;j}^{\;(i)}}{1+{F}_{r,i}+{F}_{{\rm{c}},\;j}}\right),\quad i\in [1,\ldots ,I],j\in [1,\ldots ,J]$$

where

10 $${F}_{{\rm{c}},j}=\mathop{\sum }\limits_{l=1}^{l=I}\left({D}_{l}/{K}_{lj}\right)=\mathop{\sum }\limits_{l=1}^{l=I}{F}_{{\rm{c}},j}^{\;(l)}$$

is the normalized substrate flux that describes the influence of all competing substrate fluxes towards consumer *B*_*j*_, with conjugate flux

11 $${F}_{r,i}=\mathop{\sum }\limits_{l=1}^{l=J}\left({N}_{il}{B}_{l}/{K}_{il}\right)=\mathop{\sum }\limits_{l=1}^{l=J}{F}_{r,i}^{\;(l)}$$

describing all competing consumers’ demands for a given substrate ${S}_{i},{k}_{2,ij}^{+}$ is the maximal substrate conversion rate, ${N}_{ij}=\frac{{M}_{\rm{C}}4\pi {r}_{{\rm{c}},j}^{2}{\rho }_{{\rm{porter}},ij}}{0.47{M}_{\rm{dry}}\pi {r}_{p}^{2}{N}_{\rm{A}}}$ (mol C^−1^) is the matrix of biomass-specific binding sites (with ‘radius’ *r*_*p*_), *M*_C_ is the molar mass of C, *N*_A_ is Avogrado’s constant and *K*_*ij*_ (mM) denotes the binding half-saturation constants (equation 7).

### Simulations

We simulated laboratory batch culture conditions for 39 bacterial isolates growing on 82 plant exudate metabolites. The concentration of exudates was selected to match the original growth medium concentration (125 mg C l^−^^1^), assuming that nitrogen and other essential nutrients are non-limiting for the synthesis of biomass^22^. Exudate concentrations corresponded to concentrations of dissolved organic C detected in soil excavated from the ‘Little Buck’ pasture at the University of California Hopland Research and Extension Center (38° 59’ 34.5768” N, 123° 4’ 3.7704” W), which is the traditional and ancestral territory of the Shóqowa and Hopland People, where these bacteria were originally isolated. Inocula corresponding to 10^3^ cells per gram soil were split into 90% reserve and 10% structural biomass^74^ and simulations were run for 500 h or until substrate was exhausted. The reported CUE and realized growth rates corresponded to median values averaged over the simulated growth curves. The latency of C assimilation was determined through the timing of peak growth mineralization in each simulation.

To compare maximum specific growth rates of isolates to measured growth rates in a defined growth medium (Supplementary Fig. 3 and Table 5), we defined the in silico molecular input for commercial compound mixtures (yeast extract, proteose peptone) according to manufacturer instructions, following guidelines developed for genome-scale metabolic modelling^82^.

Simulations were then extended to represent a mixed growth medium by evenly distributing the original batch exudate concentration across the different metabolites. Mixed medium simulations were run for 1,500 h or until the first metabolite was depleted, and the differences in relative uptake between rhizosphere bacterial response groups were calculated from the concentration differences at the start and end of the simulation. Using these data, resource niche breadth was calculated using Levins index^83^.

### Statistical analysis

Statistical analysis was performed using publicly available R (v.3.6.2) and Python (v.3.7.1) packages as described below.

To assess the relative proportion of variance explained by taxonomy and resource type, we used restricted maximum log-likelihood and variance partitioning analyses using the lmer function in the ‘lm4’ (v.1.1.27.1) package with default settings, followed by the r.squaredGLMM function in the ‘MuMIn’ (v.1.43.17) package. A typical analysis was coded as

$$\begin{array}{rcl}&&{\mathrm{fm}}.{\mathrm{trait}} <\!\!- \,\, {\mathrm{lmer}}({\mathrm{trait}} \sim {\mathrm{as}}.{\mathrm{factor}}({\mathrm{main}})+(1| {\mathrm{nested}}/{\mathrm{ontology}}),{\mathrm{data}})\\ &&{\mathrm{r}}.{\mathrm{squaredGLMM}}({\mathrm{fm}}.{\mathrm{trait}})\end{array}$$

and used to assess the relative importance of each variable when grouped together in a nested framework (Supplementary Table 2).

In addition, to assess the relative importance of genomic traits on isolate growth rate and C use efficiency, we used multiple regression models including rRNA operon copy number (*rrn*) and genome size (G), and the best model was selected on the basis of the smallest Akaike information criterion value. The regression analysis was performed using the lm function in R with the generic form

$$\begin{array}{rcl}&&{\mathrm{dependent}}\,{\mathrm{variable}}={\mathrm{copy}}\,{\mathrm{number}}\,{}^{* }\,{\mathrm{slope}}\,{\mathrm{rrn}}+{\mathrm{genome}}\,{\mathrm{size}}\,{}^{* }\,{\mathrm{slope}}\,{\mathrm{G}}\\ &&+{\mathrm{copy}}\,{\mathrm{number}}^{* }{\mathrm{genome}}\,{\mathrm{size}}^{* }{\mathrm{slope}}\,{\mathrm{rrn}}:{\mathrm{G}}+{\mathrm{intercept}}\end{array}$$

and results are summarized in Supplementary Table 3.

A linear regression model with growth regime (high vs low) as categorical variable was used to test for the relationship between C use efficiency and growth rate, as well as changes in BP rate due to BR rate. BP and BR were log_10_-transformed to match model assumptions (Fig. 4d). We then tested for the scaling relationship between BP and BR using a one-sample *t*-test to determine whether the slope was different from unity.

To determine model variable importance for C use efficiency and growth rate, we analysed the mean decrease in model accuracy as a measurement of the change in the accuracy of boosted random forest predictions when the variable in question was randomly permuted using the ‘gbm’ (v.2.1.8) library and within it the gbm function, as

$$\begin{array}{l}{\mathrm{fm}}.{\mathrm{boost}} <\!\!- \,\,{\mathrm{gbm}}\left.\right({\mathrm{trait}} \sim .,{\mathrm{data}}={\mathrm{train}},{\mathrm{distribution}}={}^{{\prime\prime} }{\mathrm{gaussian}}^{{\prime\prime} },\\{\mathrm{n}}.{\mathrm{trees}}=10000,{\mathrm{interaction}}.{\mathrm{depth}}=8,{\mathrm{shrinkage}}=0.001\left.\right).\end{array}$$

The number of trees, tree depth and the shrinkage parameter were selected on the basis of cross validation against the test root mean square error, using the observations for isolates originally classified as an ‘undefined’ response group as 994 out-of-bag samples (Supplementary Fig. 4).

Covariation patterns in standardized energy budget flux distributions were illustrated via a principal component analysis (PCA) using the PCA function in the ‘scikit-learn’ (v.1.02) Python package (Fig. 4e,f).

To test for significant differences in trait distributions among rhizosphere response groups, we used a Kruskal–Wallis test followed by Dunn’s test with Benjamini–Hochberg correction as implemented in the ‘FSA’ (v.0.9.2) R package.

### Reporting summary

Further information on research design is available in the Nature Portfolio Reporting Summary linked to this article.

## Supplementary information

## Data Availability

The raw experimental data can be accessed from public repositories provided in ref. ^22^. Data to reproduce the work in this paper are provided in the Supplementary Data File and registered in Zenodo at https://doi.org/10.5281/zenodo.7879221.

## Code availability

The DEBmicroTrait source code is available from the standalone modular Julia (v.1.6.7) library DEBmicroTrait.jl (https://github.com/giannamars/DEBmicroTrait.jl). All scripts to reproduce the work in this paper are registered in Zenodo at https://doi.org/10.5281/zenodo.7879221.
