# Supplementary material for: Predictions of rhizosphere microbiome dynamics with a genome-informed and trait-based energy budget model
Source: Nat Microbiol. 2024 Feb 5;9(2):421–33. doi: 10.1038/s41564-023-01582-w (PMC10847045; doi:10.1038/s41564-023-01582-w)
Supplement: Supplementary file 1 — Supplementary Tables 1–5 and Figs. 1–6. [file 41564_2023_1582_MOESM1_ESM.pdf]

nature microbiology

Article

https://doi.org/10.1038/s41564-023-01582-w

Predictions of rhizosphere microbiome dynamics with a genome-informed and trait-based energy budget model

In the format provided by the authors and unedited

Contents

Page

Suppl. Table 1: Overview of *DEBmicroTrait* trait integration. 2
Suppl. Table 2: Taxonomic and substrate variance partitioning describing the effect of isolate identity, taxonomic order, metabolite type and metabolite chemical class on carbon use efficiency. 4
Suppl. Table 3: Model selection for predicting isolate growth rates and carbon use efficiency based on rRNA copy number and genome size. 5
Suppl. Table 4: Measured and predicted half-saturation constants for substrate uptake for 13 reference genomes. 6
Suppl. Table 5: Measured, genome-predicted, and modelled minimum generation times of rhizosphere isolates. 7

Suppl. Fig. 1: Comparison of predicted half-saturation constants and measured half-saturation constants for 13 reference genomes. 8
Suppl. Fig. 2: Relationships between substrate uptake affinity and substrate binding-site density. 9
Suppl. Fig. 3: Comparison of predicted maximum specific growth rates and experimentally confirmed maximum specific growth rates of rhizosphere isolates. 10
Suppl. Fig. 4: Representation of the five most influential predictors of carbon use efficiency and growth rate in batch simulations. 11
Suppl. Fig. 5: Relationship between maximum structural biomass yield and translation power across rhizosphere isolates. 12
Suppl. Fig. 6: Benchmarking of predicted translational power of phylogenetically diverse bacteria 13

Suppl. Table 1: Overview of *DEBmicroTrait* trait integration.

Trait	Source	Formula	Unit	Source	Formula	Unit
**Life-history**	Genome-derived			Modelled		
Maximum specific growth rate	microTrait[a]	r_{max}	h^{-1}			
Genome size	microTrait/phylogeny	L_{DNA}	bp			
rRNA operon copy number	microTrait/rrnDB[b]	rrn	-			
DNA volume				Scaling law[c]	$V_{DNA} = v_N L_{DNA}$	m^3
Translation power				Biophysical model[c]	k_E	h^{-1}
Translation efficiency				Scaling law[d]	$9.5 - 1.22\log_2(\mathrm{rrn})$	-
**Biophysical**	Literature			Modelled		
Cell volume				Scaling law[c]	$V_c = (\frac{V_{DNA}}{D_0})^{1/\beta_D}$	m^3
Protein volume				Scaling law[c]	$V_P = P_0 V_c^{\beta_P}$	m^3
Average protein length	Bionumber[e] 108986	$\bar{l}_P$	bp			
Average ribosome length	Bionumber 101439	$\bar{l}_R$	bp			
Bacterial cell density				Scaling law[f]	λ_B	-
Cell stoichiometry	Biophysical model[g]	$CH_uN_vO_w$	-			
**Metabolic**	Literature			Modelled		
Ribosome volume				Biophysical model[c]	V_R	m^3
Specific ribosome degradation rate				Biophysical model[c]	$\eta = \phi$	s^{-1}
Maximum ribosome processing rate	Bionumber 100059	$\bar{r}_R$	bp s^{-1}			
Specific protein degradation rate				Biophysical model[c]	$\phi = 6.2e^{-7}$	s^{-1}
Basal maintenance rate				Scaling law[h]	$0.39V_c^{0.88}$	h^{-1}
Average transport protein cost	Estimated[i]	$1.09e^{-19}$	W			
**Stress tolerance**				Modelled		
Intrinsic mortality rate				Scaling law[j]	$\gamma_{V,0} = 0.23e^{0.88r_{max}}$	h^{-1}
Mortality half-saturation constant				Estimated	$\gamma_{V,1} = 98.8$	μM

Overview of *DEBmicroTrait* trait integration - continued.

Trait	Source	Formula	Unit	Source	Formula	Unit
**Thermodynamic**	Literature					
Reserve chemical potential	Estimated[k]	$\mu_E = 33$	kJ mol^{-1}			
Structure chemical potential	Estimated[k]	$\mu_V = 107$	kJ mol^{-1}			
**Resource acquisition**	Genome-derived			Modelled		
Membrane transport proteins	microTrait/TransportDB[l]	z_ρ	-			
Carbohydrate active enzymes	microTrait/CAZy[m]	z_X	-			
Membrane binding site density				Estimated[n]	ρ_{porter}	-
Biomass-specific site density				ECA parameter[f]	$N_{SB} = \frac{\lambda_B N_{porter}}{12.011}$	mol gC^{-1}
Maximum specific uptake rate				ECA parameter[f]	$V_{max} = k_{cat} N_{SB}$	h^{-1}
Half-saturation constant				ECA parameter[f]	K_D	μM
	Literature					
Max. substrate processing rate	Bionumber 114686	k_{cat}	s^{-1}			
**Resource use**	Modelled					
Reserve maintenance fraction	Default[o]	$y_{EM} = 1.0$	-			
Constitutive exoenzyme rate	Estimated[p]	$1e^{-2} z_X$	-			

References: a: [1], b: [2], c: [3], d: [4], e: [5], f: [6], g: [7], h: [8], i: [9], j: [10], k: [11], l: [12], m: [13], n: [14], o: [15], p: [16]

Suppl. Table 2: Taxonomic and substrate variance partitioning using linear mixed-effects models describing the effect of isolate identity, taxonomic order, metabolite type and metabolite chemical class on carbon use efficiency.

Model	Main variable	Nested variable	Variance explained (main)	Variance explained (nested)	AIC
**Taxonomic order**	Species	Metabolite	38%	88%	**-8624**
	Phylum		13%	63%	-5754
	Class		20%	69%	-6222
**Metabolite type**	Metabolite	Species	48%	88%	**-8553**
	Class		15%	54%	-5465

Mixed-effects models were fit by REML using the lmer() function in the lme4 (v1.1.27.1) R package. In these models there is a main variable and a nested variable. For each analysis, the variation explained by the main variable is accounted for before the variation explained by the nested variable is determined. As such, these results indicate the relative importance of each variable when grouped together in a nested framework.

Suppl. Table 3: Model selection for predicting isolate growth rates and carbon use efficiency based on rRNA copy number (rrn) and genome size (G). The predictions were split based on growth rate into a high (>0.041 h^{-1}) vs. low (<0.04 h^{-1}) growth regime.

		Model	Slope rrn	Slope G	Intercept	p-value rrn	p-value G	p-value rrn:G	r^2	AIC
**Growth rate**										
	High	rrn	0.0125		0.0530	<2e-16		<2e-16	0.30	**-4997**
		G		-1.8e-8	0.165		<2e-16	<2e-16	0.23	-4847
		rrn:G	0.0131	1.1e-9	0.0459	<2e-16	0.503	<2e-16	0.30	-4996
	Low	rrn	0.00117		0.0168	4.53e-8		1.2e-10	0.04	**-8427**
		G		-8.3e-10	0.0228		0.00015	0.13467	0.02	-8411
		rrn:G	0.00124	1.0e-10	0.0162	7.7e-5	0.749	2.9e-5	0.04	-8425
**Carbon use efficiency**										
	High	rrn	-0.015		0.628	<2e-16		0.641	0.44	**-3089**
		G		2.2e-8	0.488		<2e-16	<2e-16	0.42	-3051
		rrn:G	-0.013	3.7e-9	0.605	3.7e-10	0.263	0.44	0.44	-3088
	Low	rrn	-0.018		0.582	4.0e-14		0.807	0.35	-1952
		G		9.6e-9	0.505		0.000127	< 2e-16	0.32	-1909
		rrn:G	-0.025	-9.2e-9	0.636	2.8e-12	0.0117	0.0634	0.35	**-1956**

All models have the generic form: Dependent variable = copy number * slope rrn+genome size * slope G + copy number*genome size *slope rrn:G+intercept. Blank cells indicate cases where a term was excluded from the model (e.g., a model based on rrn, rRNA copy number, will not have a slope or p-value estimate for G, genome size). For cases where at least one model was statistically significant (two-sided p-value < .05) , the best model based on the smallest AIC value is indicated in bold.

Suppl. Table 4: Measured and predicted half-saturation constants for substrate uptake for 13 reference genomes as reported in [29].

Substrate	Taxonomy	Strain	Method, Incubation time	g_{max} [h^{-1}]	V_{max} [h^{-1}]	K_{meas} [μM]	K_{model} [μM]	
Glucose	Flavobacterium johnsoniae	C-21	Steady-state in continuous culture	0.2	[-]	1.55	1.89	[17]
Glucose	Escherichia coli	ML 308	Batch culture growth	1.238	[-]	13.0	13.93	[18]
Lactose	Lacticaseibacillus casei	64 H	Radioactivity uptake, 10 min	[-]	2.19	14.0	15.53	[19]
Glucose	Corynebacterium sp.	198	$^{14}CO_2$ from continuous culture samples	0.15	[-]	0.48	0.39	[20]
Tyrosine	Brevibacterium linens	47	Initial uptake, 5 min	[-]	1.05	3.40	3.78	[21]
Phenylalanine	Brevibacterium linens	47	Initial uptake, 5 min	[-]	1.75	25.0	10.7	[21]
Tryptophan	Brevibacterium linens	[-]	Initial uptake, 5 min	[-]	0.35	1.8	0.78	[21]
Isoleucine	Streptococcus thermophylus	302	Radioactivity uptake, 1 min	[-]	0.95	36	32	[22]
Valine	Streptococcus thermophylus	302	Radioactivity uptake, 1 min	[-]	1.20	2.0	1.3	[22]
Glycerol-3-phosphate	Escherichia coli	[-]	Radioactivity uptake, 1 min	[-]	0.006	2.0	1.03	[23]
Succinate	Rhizobium leguminosarum	[-]	Radioactivity uptake, 2 min	[-]	0.06	2.0	1.3	[24]
Toluene	Pseudomonas sp	[-]	Total ^{14}C-labelled product production, 4 hrs	[-]	0.111	0.47	0.65	[25]
Methanol	Pseudomonas sp	MA	Radioactivity uptake, 10 min	[-]	0.05	4.8	2.78	[26]
Fructose	Thiobacillus sp.	A2	Dyalisis rate of ^{14}C labelled substrate, 5 min	0.39	0.68	410	1.86	[27]
Ribose	Thiobacillus sp.	A3	Dyalisis rate of ^{14}C labelled substrate, 5 min	0.03	0.02	69	0.86	[27]
Succinate	Bradyrhizobium japonicum	[-]	Radioactivity uptake, 10 min	[-]	0.001	1.8	0.17	[28]

Abbreviations: maximum specific growth rate: g_{max} [h^{-1}], maximum specific uptake rate: V_{max} [h^{-1}], measured half-saturation constant: K_{meas} [μM], estimated half-saturation constant: K_{model} [μM]. Literature maximum uptake rates reported in units of nmol/min/mg were converted to the given unit by scaling with estimates of cellular dry mass [3].

Suppl. Table 5: Measured, genome-predicted, and modelled minimum generation times (minGT) of rhizosphere isolates.

Isolate (Abbr.)	Classification	minGT [h] measured	minGT [h] predicted	minGT [h] modelled
HE68	positive	-	7.00	6.43
HB09	negative	-	1.31	1.50
HD36	undefined	1.55	2.78	2.93
HA02	positive	6.05	6.09	8.94
HA13	positive	5.9	5.73	6.63
HD69	positive	5.93	5.32	6.58
HA54	positive	2.31	2.43	2.48
HA33	positive	-	2.90	2.97
HD24	undefined	3.57	2.52	2.20
HE23	undefined	2.34	3.57	2.90
HA28	positive	3.1	6.24	13.03
HE60	positive	5.96	6.09	5.64
HB58	undefined	3.59	3.33	3.08
HA31	negative	3.28	2.72	2.54
HB48	negative	4.67	3.60	2.92
HB62	negative	3.62	2.24	2.00
HB13	undefined	2.55	2.25	2.11
HA19	undefined	1.87	5.26	5.77
HB36	undefined	3.99	3.88	3.67
HA56	positive	4.25	4.70	4.28
HB07	positive	5.58	5.01	4.57
HC08	positive	5.07	5.12	4.45
HA36	undefined	2.45	1.82	1.57
HE70	negative	-	4.16	4.23
HB44	positive	4.34	4.36	4.22
HD25	positive	3.87	3.83	4.07
HA20	undefined	5.41	6.28	5.31
HA32	undefined	4.44	4.82	3.87
HA14	negative	-	1.43	2.95
HB15	positive	2.52	0.96	1.59
HD17	positive	-	7.05	8.46
HD88	undefined	3.69	7.49	7.94
HD82	negative	4.95	6.11	5.29
HD59	negative	6.29	4.74	4.30
HD07	positive	6.22	6.30	6.33
HD57	positive	-	6.78	6.20
HA57	undefined	-	0.75	0.81
HA41	positive	-	4.04	3.62
HB20	positive	7.13	8.33	10.17

Measured and genome-predicted minimum generation times as originally reported in [30].

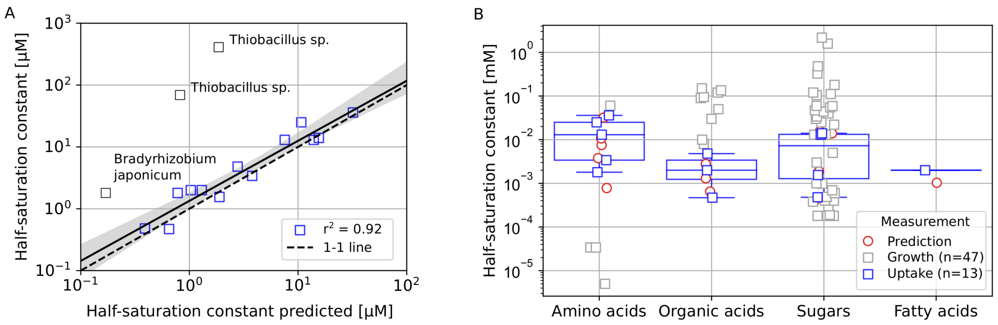

Suppl. Fig. 1: **A** Comparison of predicted half-saturation constants and measured half-saturation constants for 13 reference genomes as reported in [29]. The solid line indicates the regression line excluding outliers (r^2=0.92), while shaded areas indicate the 95% confidence bands. Data points that were excluded from the regression are labelled by species name. The dashed line corresponds to the 1-1 line for log-scaled observed vs. predicted values. **B** Survey of literature-derived half-saturation constants for different substrate classes. Each point represents a different measurement or model prediction; color indicates whether a measurement corresponds to model predictions (red), or reflects either true uptake kinetics (blue, n=13) or growth kinetics (gray, n=47). Each boxplot corresponds to measured half-saturation constants for substrate uptake. The top and bottom of each box represent the 25th and 75th percentiles, the horizontal line inside each box represents the median and the whiskers represent the range of points. The p-values below indicate statistical differences in the literature-derived and genome-predicted half-saturation constants for different substrate classes as determined by a Kruskal-Wallis test: amino acids (n=9, p=0.74), organic acids (n=12, p=0.04), sugars (n=39, p=0.50), fatty acids (n=1, p=N/A).

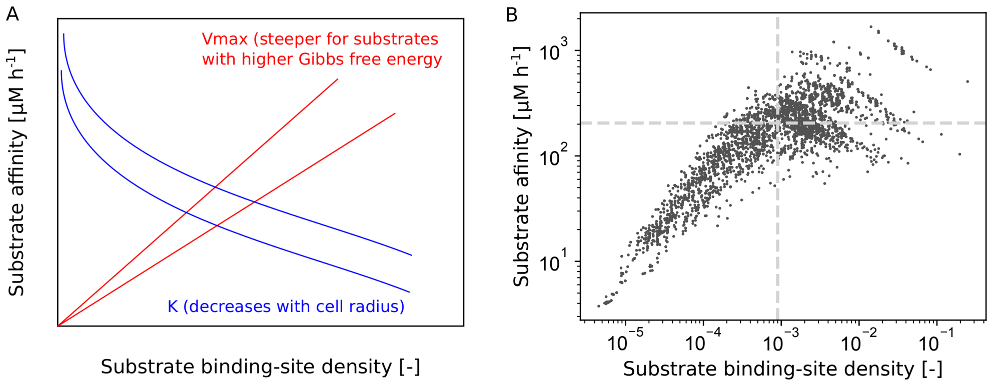

Suppl. Fig. 2: **A** Relationship between the substrate uptake affinity at low external substrate concentration ($J_{S\to 0} = V_{max}/K$) and substrate binding-site density in the equilibrium chemistry approximation (ECA [6]) for substrate uptake. **B** Estimated substrate affinity as a function of the substrate binding-site density of rhizosphere isolates. The dashed lines indicate the substrate binding-site density corresponding to the locally weighted maximum substrate affinity. Substrates: n=82, consumers: n=39.

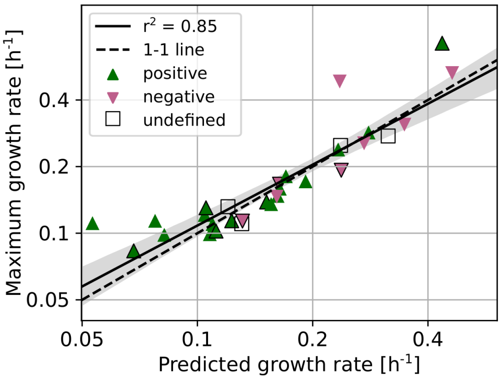

Suppl. Fig. 3: Comparison of predicted maximum specific growth rates of rhizosphere isolates (n=39) and confirmed genome-predicted maximum specific growth rates through laboratory growth rate experiments [30]. Isolates are colored by their response to plant growth (green: positive, magenta: negative, white: undefined). Following guidelines developed for genome-scale models [31], the in-silico growth medium was designed to match the original R2 1/10 medium, on which the isolates were originally cultured at 28 °C. The solid line indicates the regression line (r^2=0.85), while shaded areas indicate the 95% confidence bands. The dashed line corresponds to the 1-1 line for observed vs. predicted values.

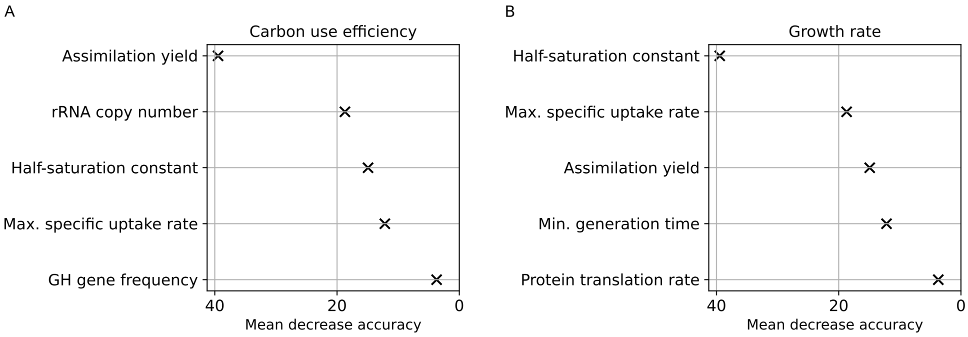

Suppl. Fig. 4: Representation of the five most influential predictors of carbon use efficiency (**A**) and growth rate (**B**) in batch simulations as determined by mean decrease in accuracy. The mean decrease in accuracy is a measurement of the change in the accuracy of the random forest's predictions when the variable in question is randomly permuted. Labels on the y axis indicate the feature names. Feature contributions for all case studies were computed on predictions for the *undefined* rhizosphere isolate response group as out-of-bag samples.

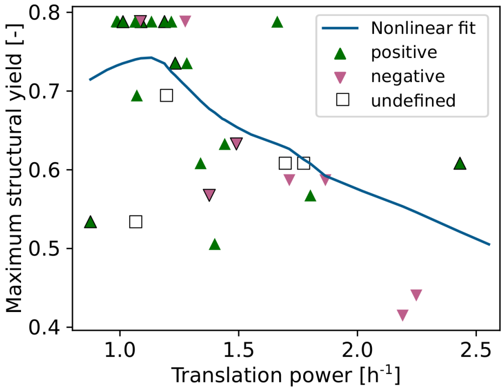

Suppl. Fig. 5: Relationship between maximum structural biomass yield and translation power across rhizosphere isolates (n=39). Isolates are colored by their response to plant growth (green: positive, magenta: negative, white: undefined). The blue line corresponds to a locally weighted linear regression model.

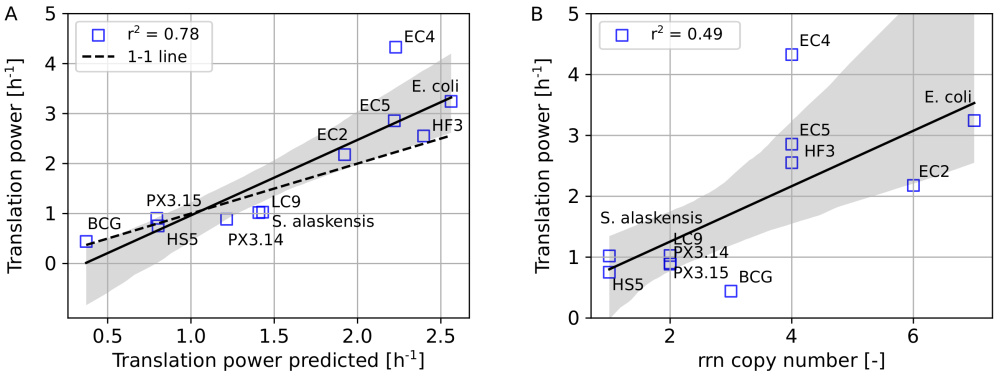

Suppl. Fig. 6: **A** Regression scatter plot for translational power of phylogenetically diverse bacteria as reported in [4, 32] (r^2=0.78). In **A** and **B**, the solid line indicates the regression line, while shaded areas indicate the 95% confidence bands. The dashed line corresponds to the 1-1 line for observed vs. predicted values (n=11). **B** Correlation between translation power and $\log_2$ rrn operon copy number (r^2=0.49). Species are the same as in **A**.

References

[1] Karaoz, U. & Brodie, E. L. microtrait: a toolset for a trait-based representation of microbial genomes. *Frontiers in Bioinformatics* **2** (2022).

[2] Stoddard, S. F., Smith, B. J., Hein, R., Roller, B. R. & Schmidt, T. M. rrn DB: improved tools for interpreting rRNA gene abundance in bacteria and archaea and a new foundation for future development. *Nucleic Acids Research* **43**, D593–D598 (2015).

[3] Kempes, C. P., Wang, L., Amend, J. P., Doyle, J. & Hoehler, T. Evolutionary tradeoffs in cellular composition across diverse bacteria. *The ISME Journal* **10**, 2145–2157 (2016).

[4] Roller, B. R., Stoddard, S. F. & Schmidt, T. M. Exploiting rRNA operon copy number to investigate bacterial reproductive strategies. *Nature Microbiology* **1**, 1–7 (2016).

[5] Milo, R., Jorgensen, P., Moran, U., Weber, G. & Springer, M. BioNumbers–the database of key numbers in molecular and cell biology. *Nucleic Acids Research* **38**, D750–D753 (2010).

[6] Tang, J. & Riley, W. J. Competitor and substrate sizes and diffusion together define enzymatic depolymerization and microbial substrate uptake rates. *Soil Biology and Biochemistry* **139**, 107624 (2019).

[7] Vrede, T., Dobberfuhl, D. R., Kooijman, S. & Elser, J. J. Fundamental connections among organism C:N:P stoichiometry, macromolecular composition, and growth. *Ecology* **85**, 1217–1229 (2004).

[8] Lynch, M. & Marinov, G. K. The bioenergetic costs of a gene. *Proceedings of the National Academy of Sciences* **112**, 15690–15695 (2015).

[9] Kempes, C. P., Koehl, M. & West, G. B. The scales that limit: the physical boundaries of evolution. *Frontiers in Ecology and Evolution* **7**, 242 (2019).

[10] Biselli, E., Schink, S. J. & Gerland, U. Slower growth of escherichia coli leads to longer survival in carbon starvation due to a decrease in the maintenance rate. *Molecular Systems Biology* **16**, e9478 (2020).

[11] Sousa, T., Mota, R., Domingos, T. & Kooijman, S. M. Thermodynamics of organisms in the context of dynamic energy budget theory. *Physical Review E* **74**, 051901 (2006).

[12] Elbourne, L. D., Tetu, S. G., Hassan, K. A. & Paulsen, I. T. TransportDB 2.0: a database for exploring membrane transporters in sequenced genomes from all domains of life. *Nucleic Acids Research* **45**, D320–D324 (2017).

[13] Drula, E. *et al.* The carbohydrate-active enzyme database: functions and literature. *Nucleic Acids Research* **50**, D571–D577 (2022).

[14] Flynn, K. J., Skibinski, D. O. & Lindemann, C. Effects of growth rate, cell size, motion, and elemental stoichiometry on nutrient transport kinetics. *PLoS Computational Biology* **14**, e1006118 (2018).

[15] Tolla, C., Kooijman, S. A. & Poggiale, J.-C. A kinetic inhibition mechanism for maintenance. *Journal of Theoretical Biology* **244**, 576–587 (2007).

[16] Traving, S. J., Thygesen, U. H., Riemann, L. & Stedmon, C. A. A model of extracellular enzymes in free-living microbes: which strategy pays off? *Applied and Environmental Microbiology* **81**, 7385–7393 (2015).

[17] Höfle, M. G. Glucose uptake of cytophaga johnsonae studied in batch and chemostat culture. *Archives of Microbiology* **133**, 289–294 (1982).

[18] Koch, A. L. & Houston Wang, C. How close to the theoretical diffusion limit do bacterial uptake systems function? *Archives of Microbiology* **131**, 36–42 (1982).

[19] Chassy, B. & Thompson, J. Regulation of lactose-phosphoenolpyruvate-dependent phosphotransferase system and beta-D-phosphogalactoside galactohydrolase activities in lactobacillus casei. *Journal of Bacteriology* **154**, 1195–1203 (1983).

[20] Law, A. & Button, D. Multiple carbon source-limited growth kinetics of a marine coryneform bacterium. *Journal of Bacteriology* **129**, 115–123 (1977).

[21] Boyaval, P., Moreira, E. & Desmazeaud, M. Transport of aromatic amino acids by Brevibacterium linens. *Journal of Bacteriology* **155**, 1123–1129 (1983).

[22] Akpemado, K. & Bracquart, P. Uptake of branched-chain amino acids by Streptococcus thermophilus. *Applied and Environmental Microbiology* **45**, 136–140 (1983).

[23] Schweizer, H., Argast, M. & Boos, W. Characteristics of a binding protein-dependent transport system for sn-glycerol-3-phosphate in Escherichia coli that is part of the pho regulon. *Journal of Bacteriology* **150**, 1154–1163 (1982).

[24] Finan, T., Wood, J. & Jordan, D. C. Symbiotic properties of C4-dicarboxylic acid transport mutants of Rhizobium leguminosarum. *Journal of Bacteriology* **154**, 1403–1413 (1983).

[25] Robertson, B. & Button, D. Toluene induction and uptake kinetics and their inclusion in the specific-affinity relationship for describing rates of hydrocarbon metabolism. *Applied and Environmental Microbiology* **53**, 2193–2205 (1987).

[26] Bellion, E., Kent, M. E., Aud, J. C., Alikhan, M. & Bolbot, J. A. Uptake of methylamine and methanol by Pseudomonas sp. strain am1. *Journal of Bacteriology* **154**, 1168–1173 (1983).

[27] Wood, A. P. & Kelly, D. P. Kinetics of sugar transport by Thiobacillus a2. *Archives of Microbiology* **131**, 156–159 (1982).

[28] McAllister, C. F. & Lepo, J. Succinate transport by free-living forms of Rhizobium japonicum. *Journal of Bacteriology* **153**, 1155–1162 (1983).

[29] Button, D. Kinetics of nutrient-limited transport and microbial growth. *Microbiological Reviews* **49**, 270–297 (1985).

[30] Zhalnina, K. *et al.* Dynamic root exudate chemistry and microbial substrate preferences drive patterns in rhizosphere microbial community assembly. *Nature Microbiology* **3**, 470–480 (2018).

[31] Marinos, G., Kaleta, C. & Waschina, S. Defining the nutritional input for genome-scale metabolic models: A roadmap. *PLoS One* **15**, e0236890 (2020).

[32] Dethlefsen, L. & Schmidt, T. M. Performance of the translational apparatus varies with the ecological strategies of bacteria. *Journal of Bacteriology* **189**, 3237–3245 (2007).
